# Supplementary material for: GeneCompete: an integrative tool of a novel union algorithm with various ranking techniques for multiple gene expression data
Source: PeerJ Comput Sci. 2023 Nov 15;9:e1686. doi: 10.7717/peerj-cs.1686 (PMC10703088; doi:10.7717/peerj-cs.1686)
Supplement: Supplemental Information 1 [file peerj-cs-09-1686-s001.docx]

**Data S1** The derivation of Colley’s rating

Let *r_i_* be the Colley’s rating.

First, the winning percentage based on Laplacian’s rule is defined by $r_{i}=\frac{W_{i}+1}{N_{i}+2}$.

Since all teams begin with *r_k_*=1/2, then the number of times player *i* win can be written as

$$W_{i}=\frac{W_{i}-L_{i}}{2}+\frac{W_{i}+L_{i}}{2}=\frac{W_{i}-L_{i}}{2}+\frac{N_{i}}{2}=\frac{W_{i}-L_{i}}{2}+\sum_{k=1}^{N_{i}} \frac{1}{2}\approx\frac{W_{i}-L_{i}}{2}+\sum_{k\in O_{i}} r_{k}$$

where *O_i_* is the set of opponents of player *i*.

After substitute W_i_,

$$r_{i}=\frac{W_{i}+1}{N_{i}+2}=\frac{\frac{W_{i}-L_{i}}{2}+\sum_{k\in O_{i}} r_{k}+1}{N_{i}+2}.$$

Then, multiplying by N_i_+2,

$${(N}_{i}+2)r_{i}=\frac{W_{i}-L_{i}}{2}+\sum_{k\in O_{i}} r_{k}+1.$$

Next, $\sum_{k\in O_{i}} r_{k}$ is subtracted from both sides of equation to obtain,

$${(N}_{i}+2)r_{i}-\sum_{k\in O_{i}} r_{k}=\frac{W_{i}-L_{i}}{2}+1.$$

Finally, *Cr=b* is obtained.
