# Supplementary figures and images for: GeneCompete: an integrative tool of a novel union algorithm with various ranking techniques for multiple gene expression data
Source: PeerJ Comput Sci. 2023 Nov 15;9:e1686. doi: 10.7717/peerj-cs.1686 (PMC10703088; doi:10.7717/peerj-cs.1686)

## Classic

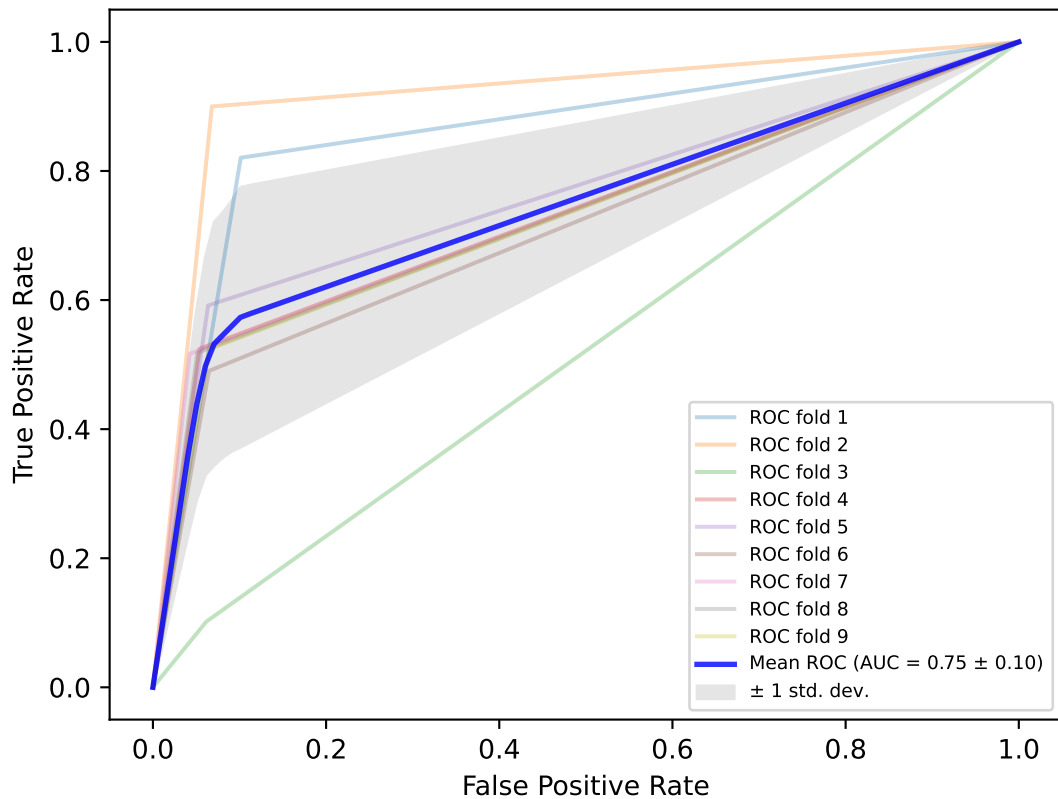

Count

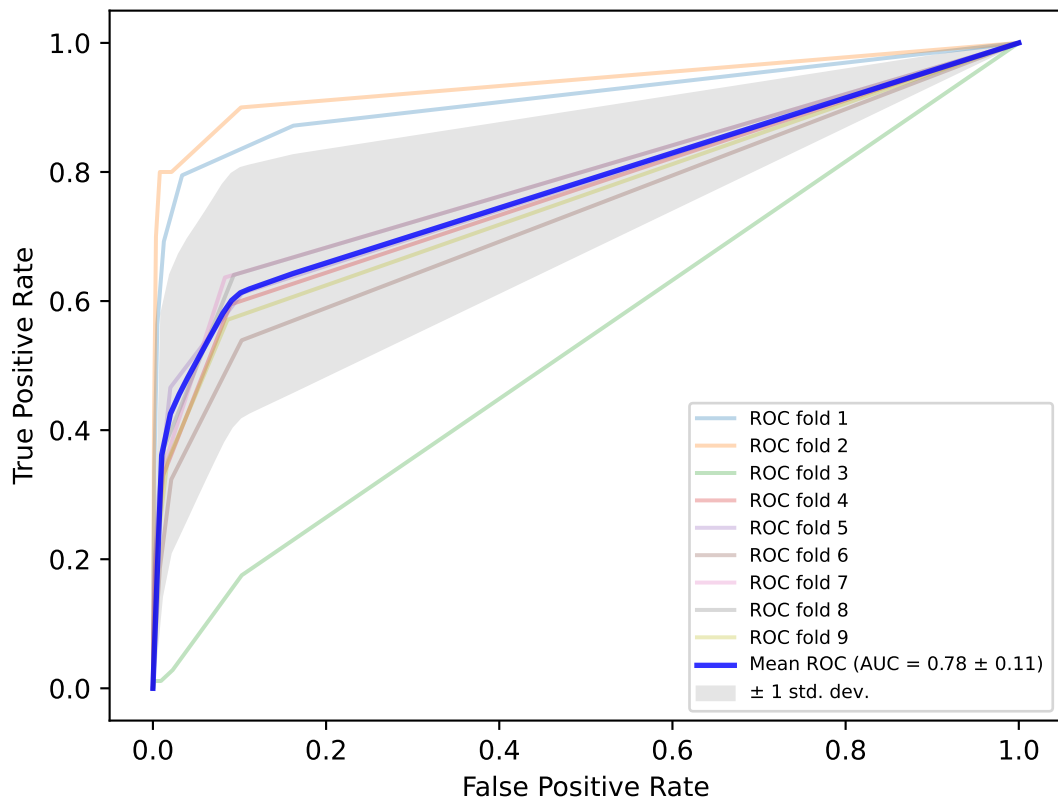

Win-loss

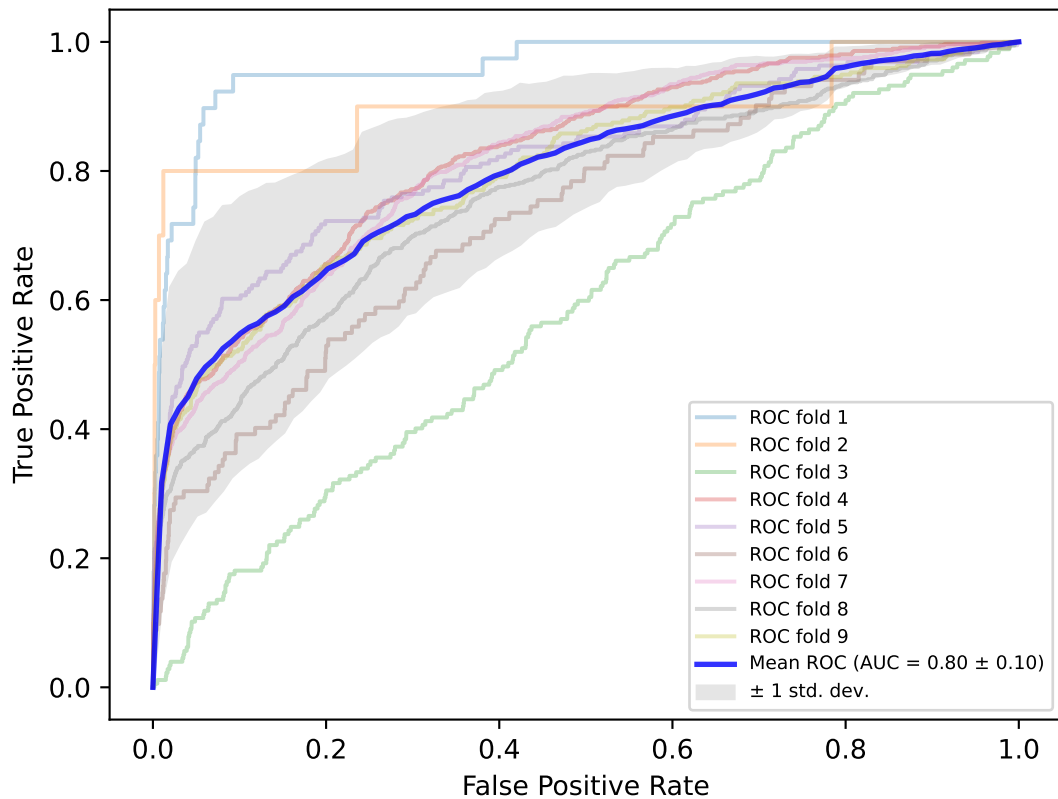

## Massey

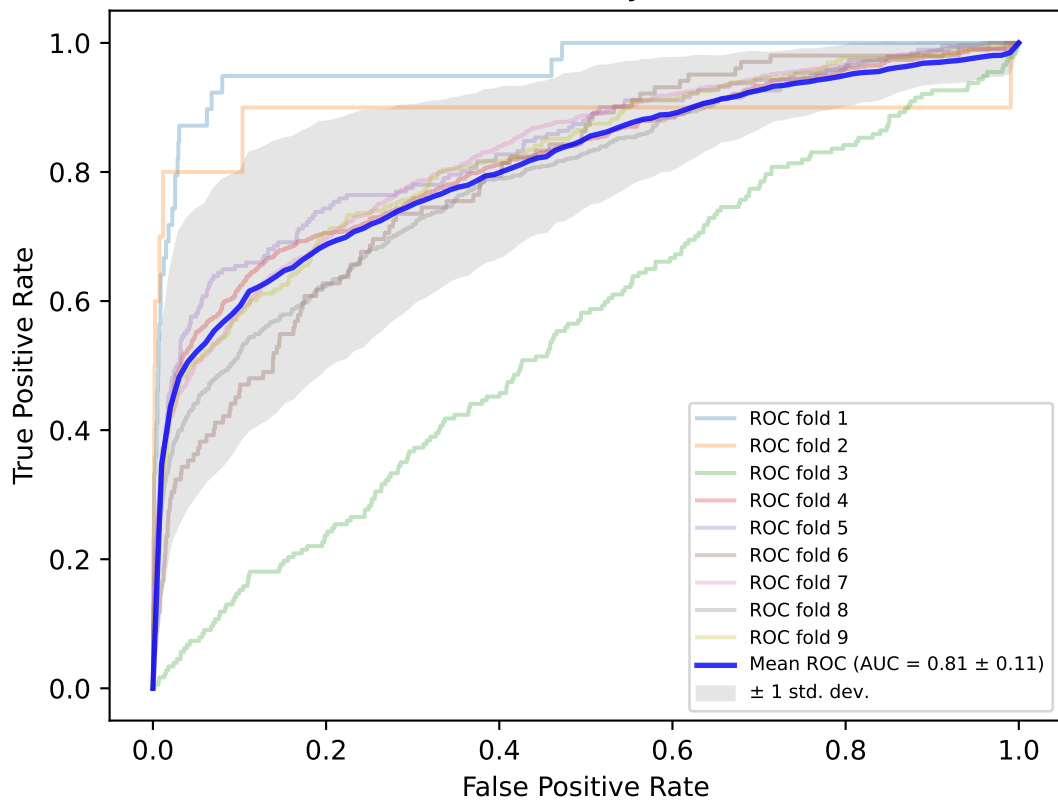

## Colley

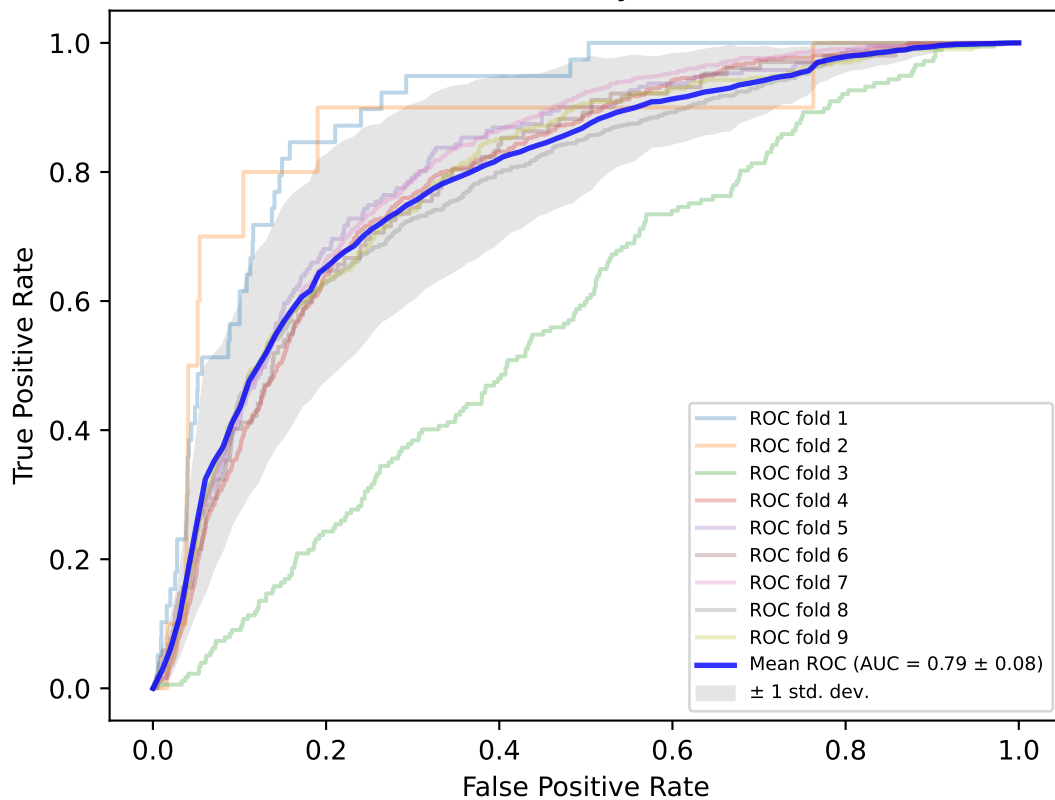

## Keener

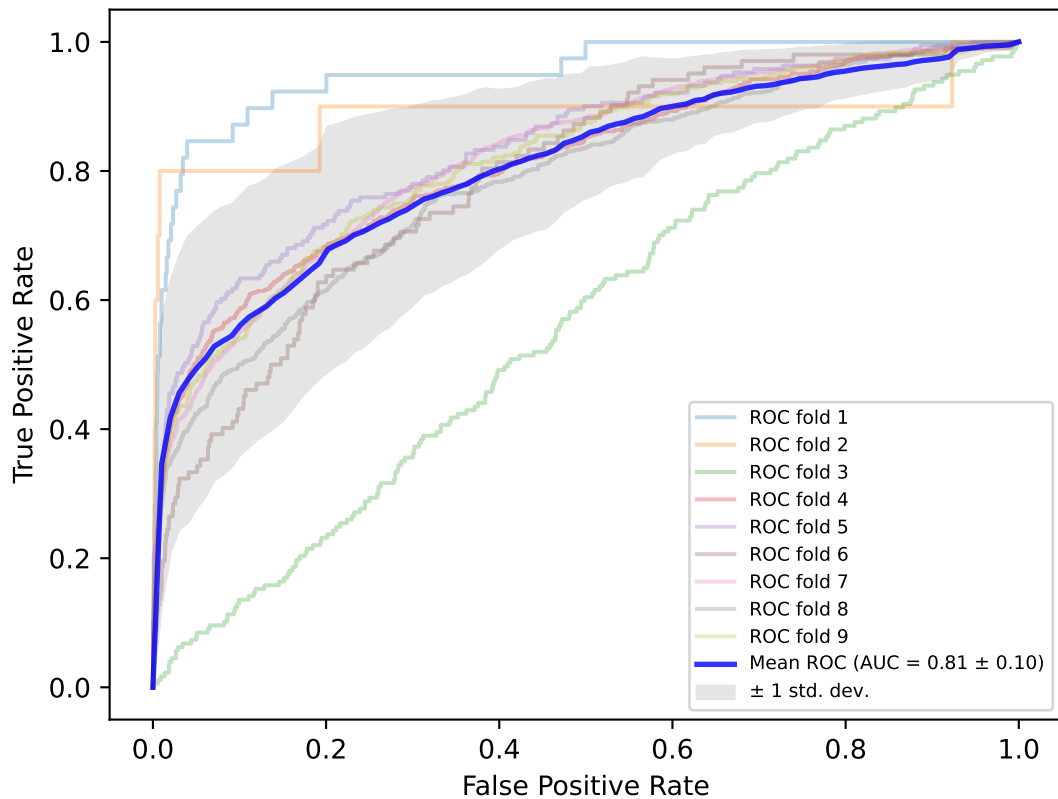

Elo

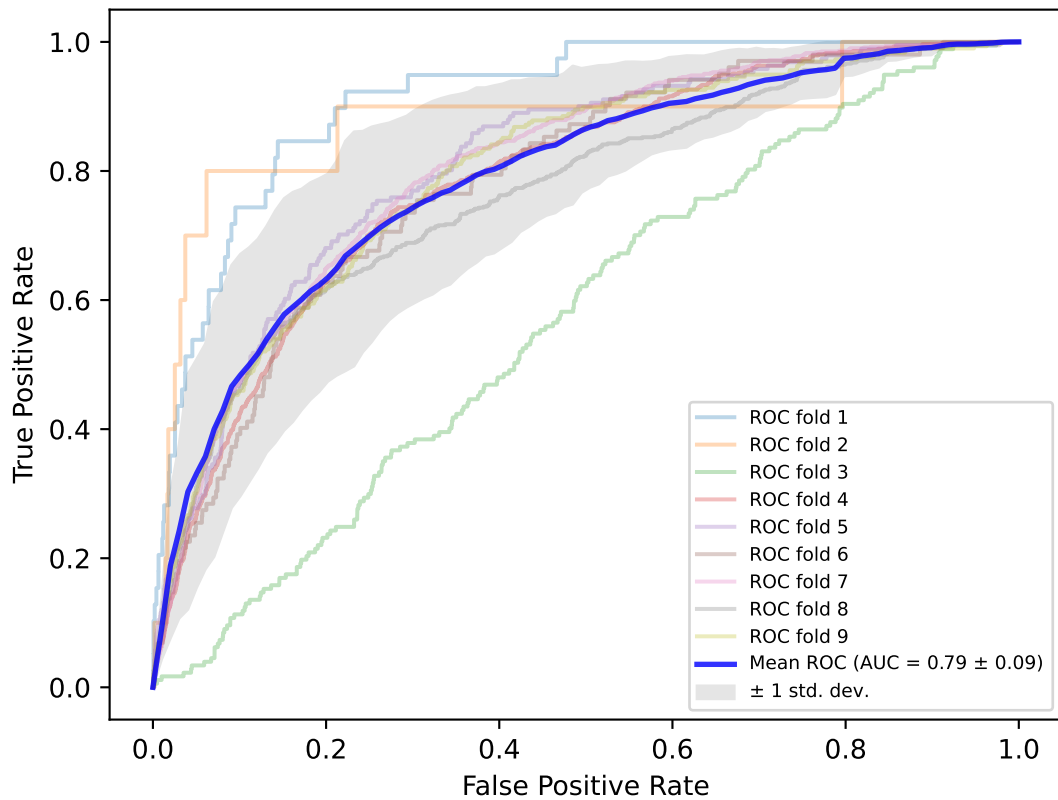

## Markov

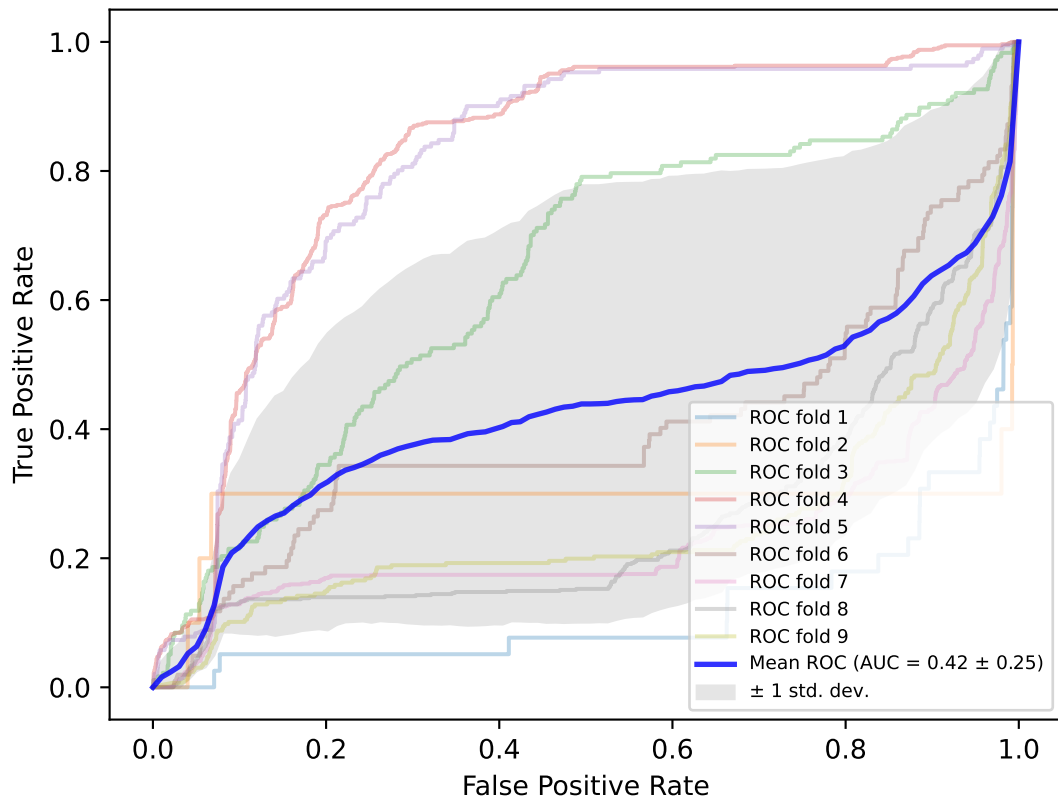

## PageRank

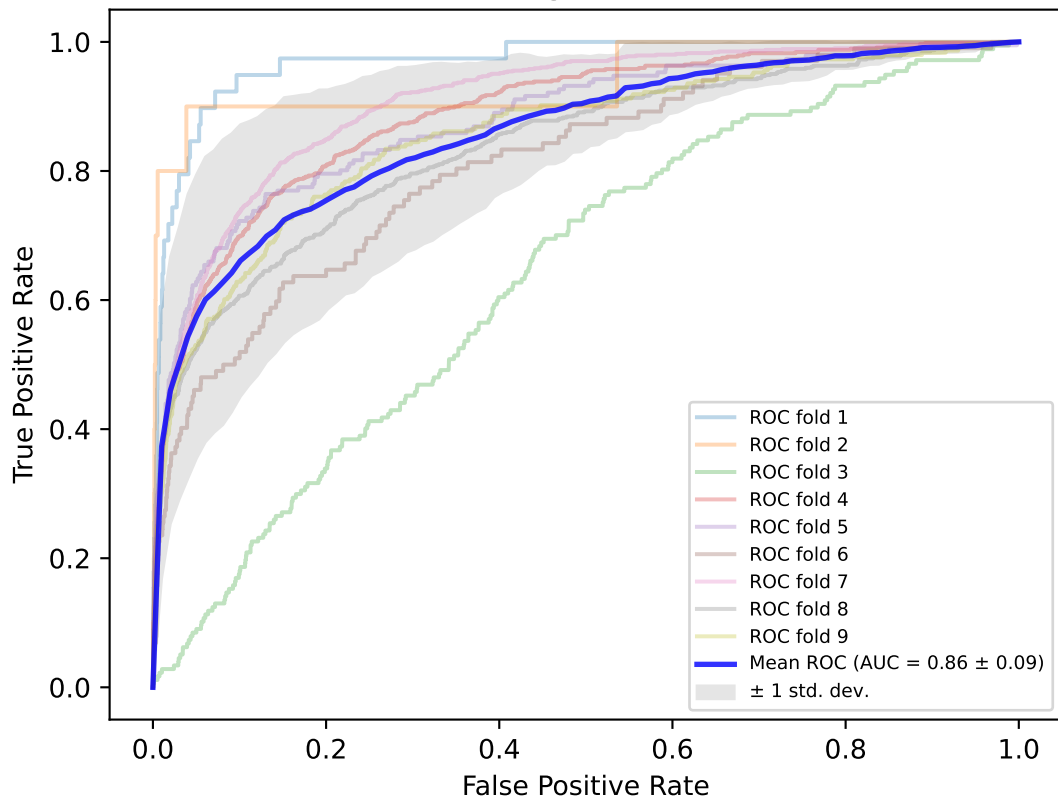

## BiPageRank

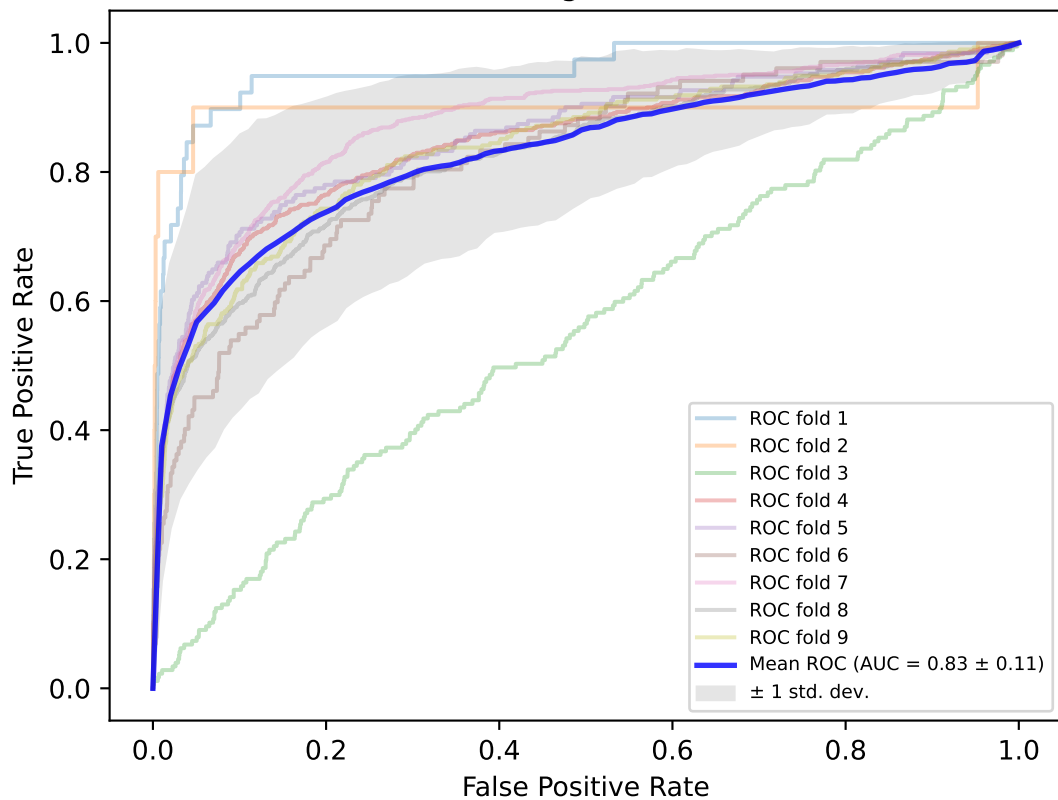

Supplement: Supplemental Information 2 [file peerj-cs-09-1686-s002.pdf]

## Classic

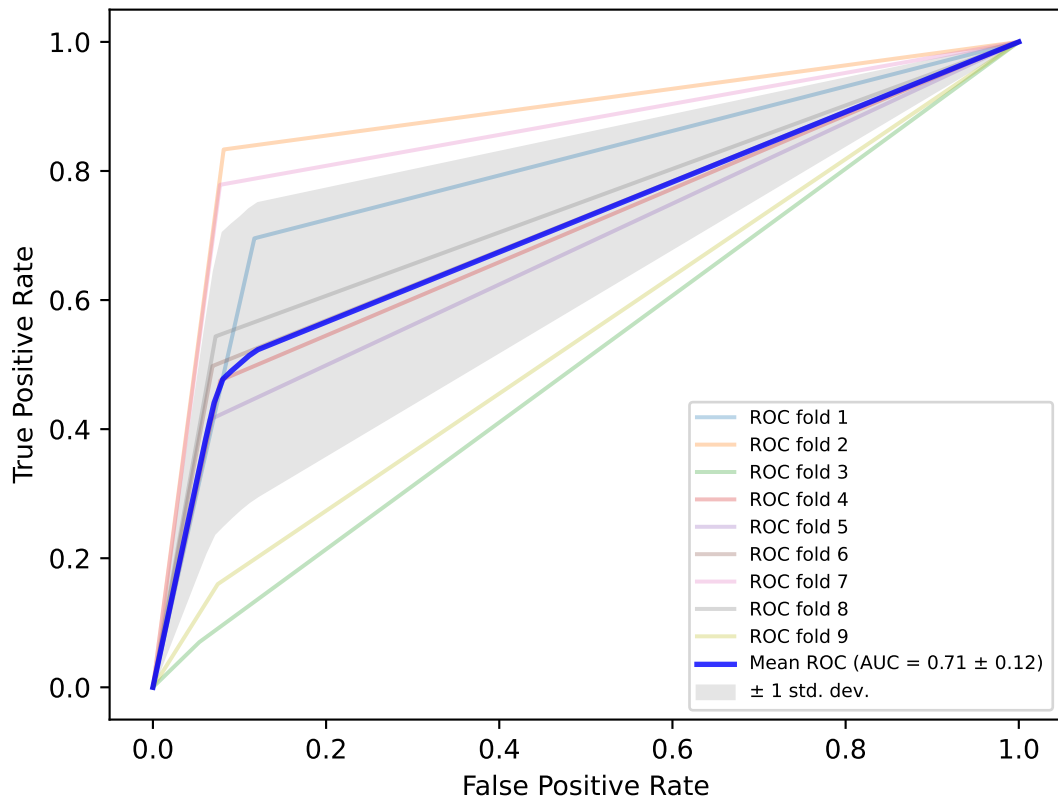

Count

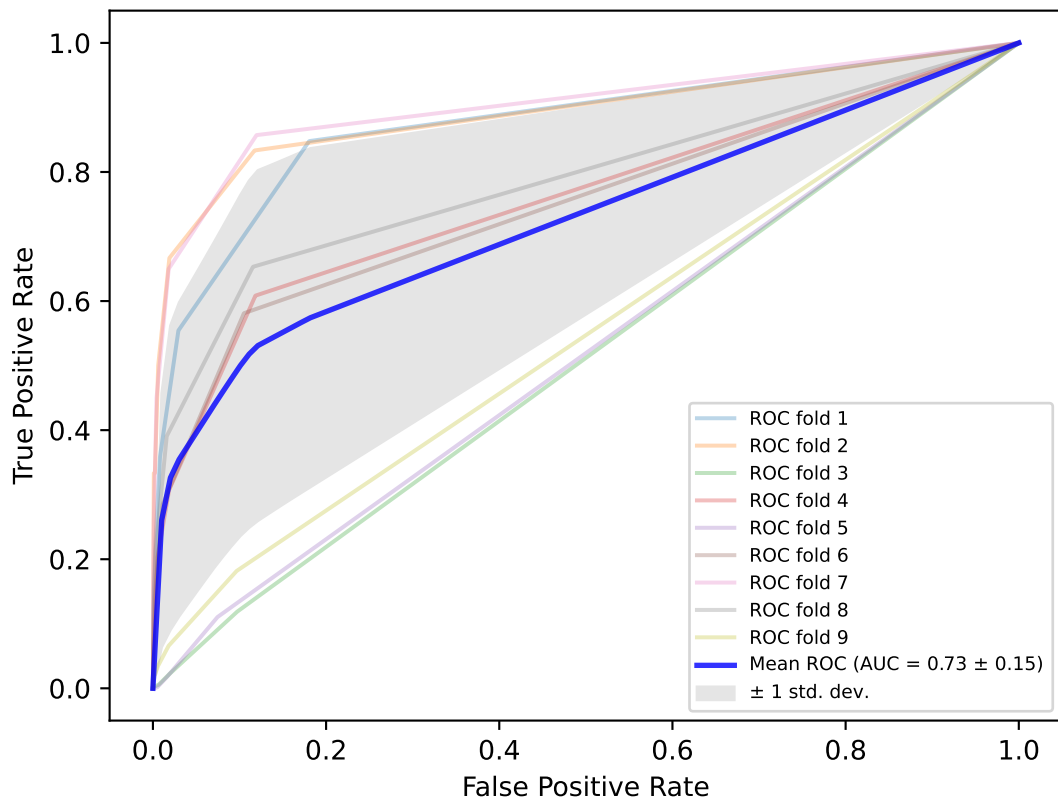

Win-loss

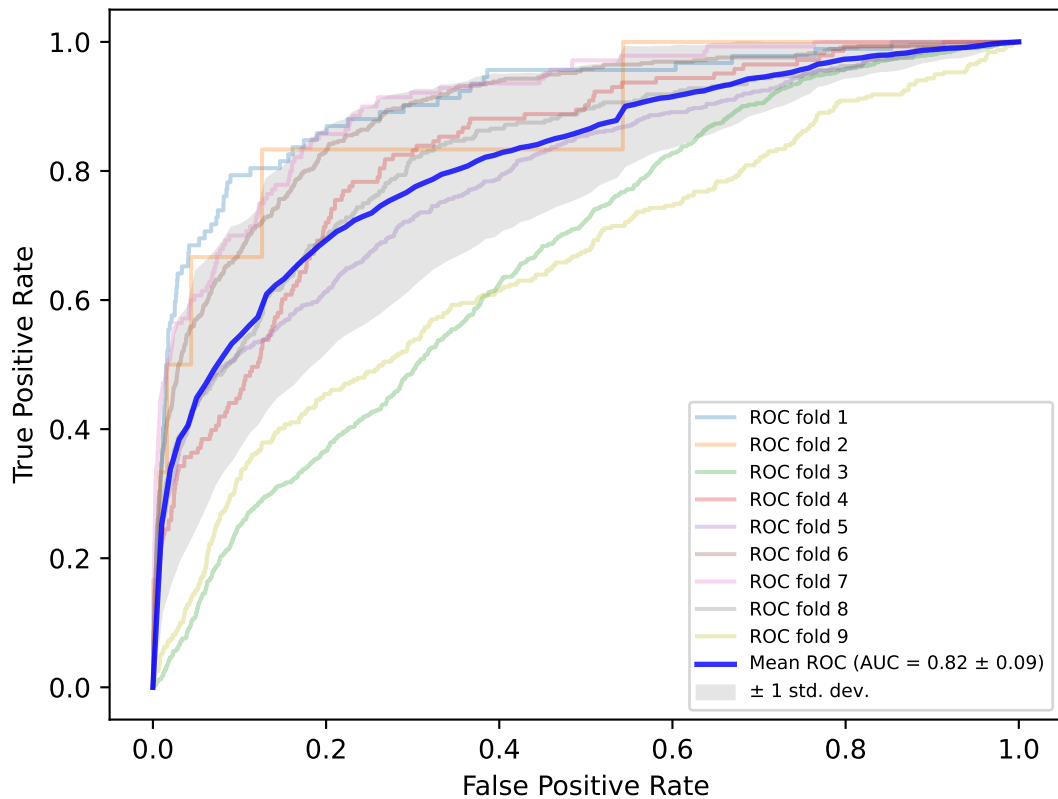

## Massey

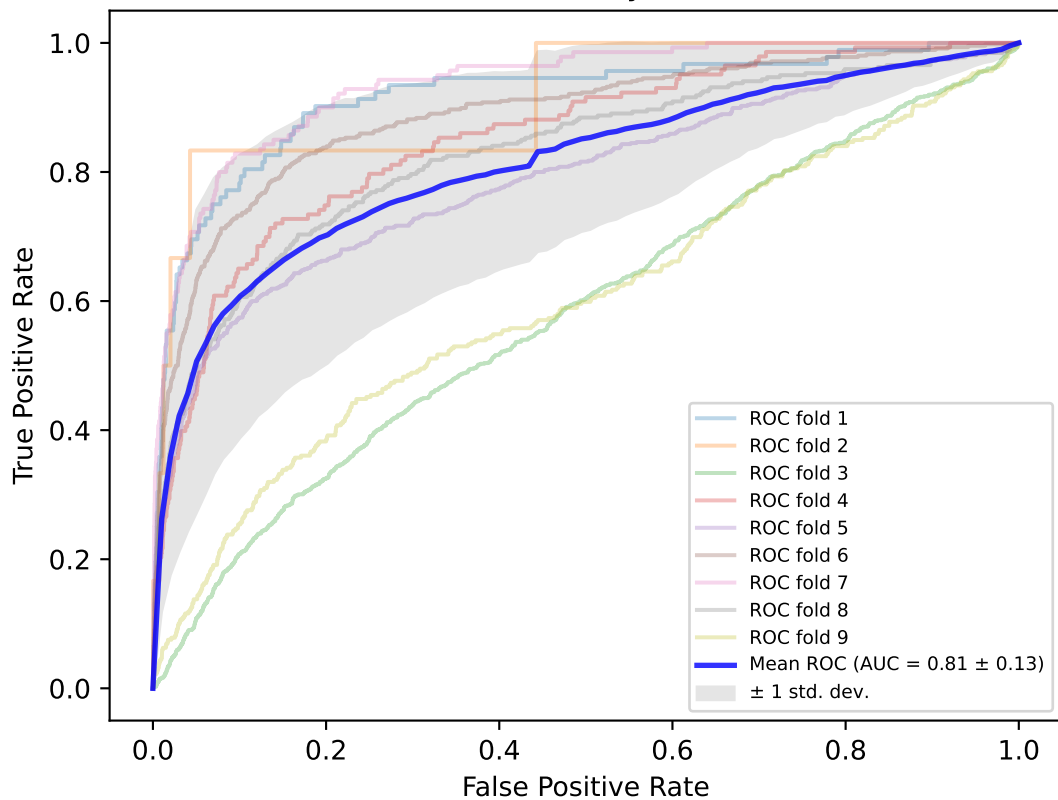

## Colley

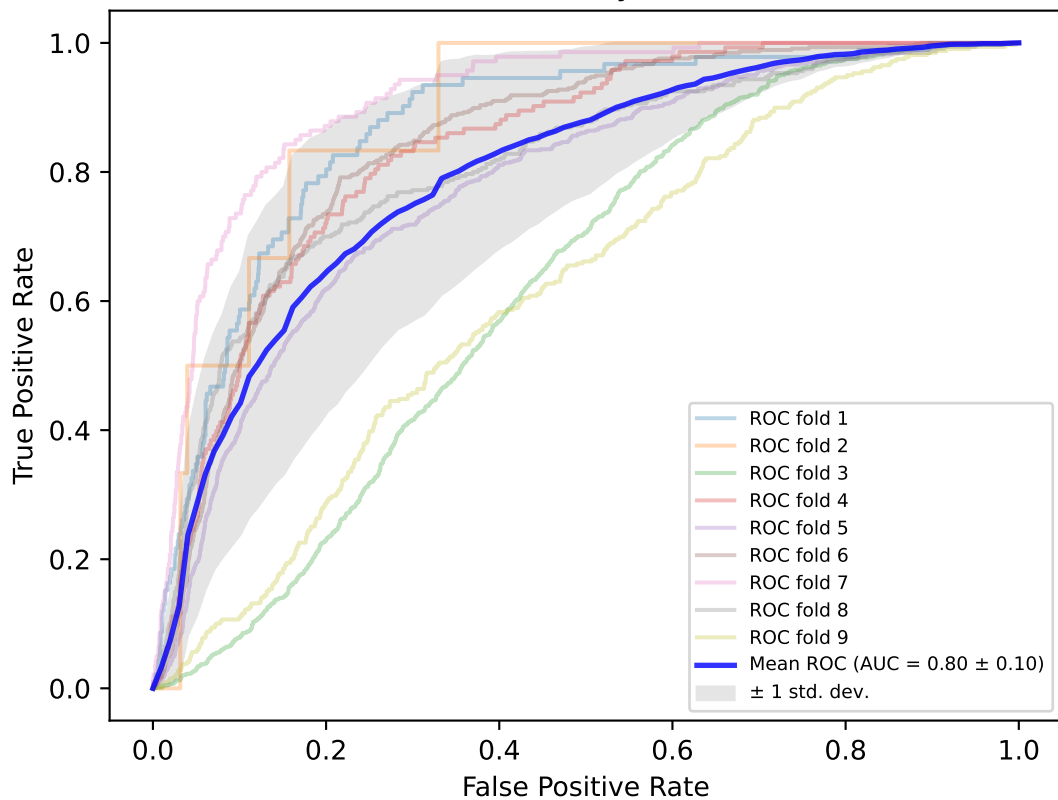

## Keener

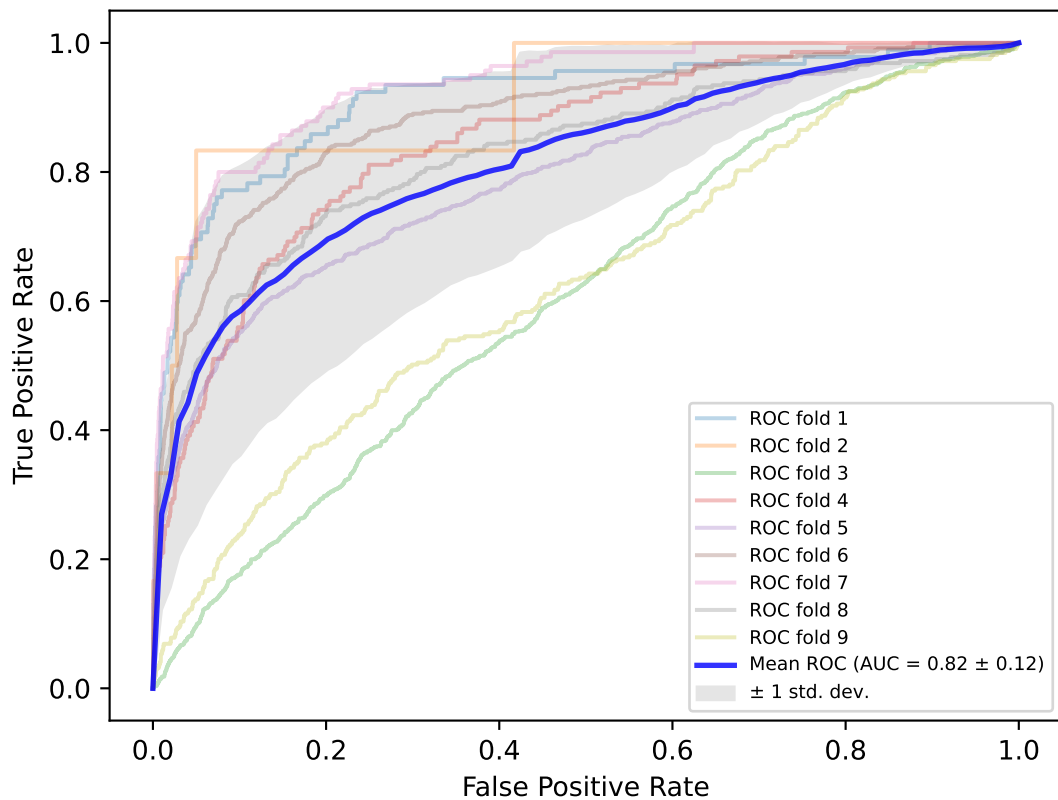

Elo

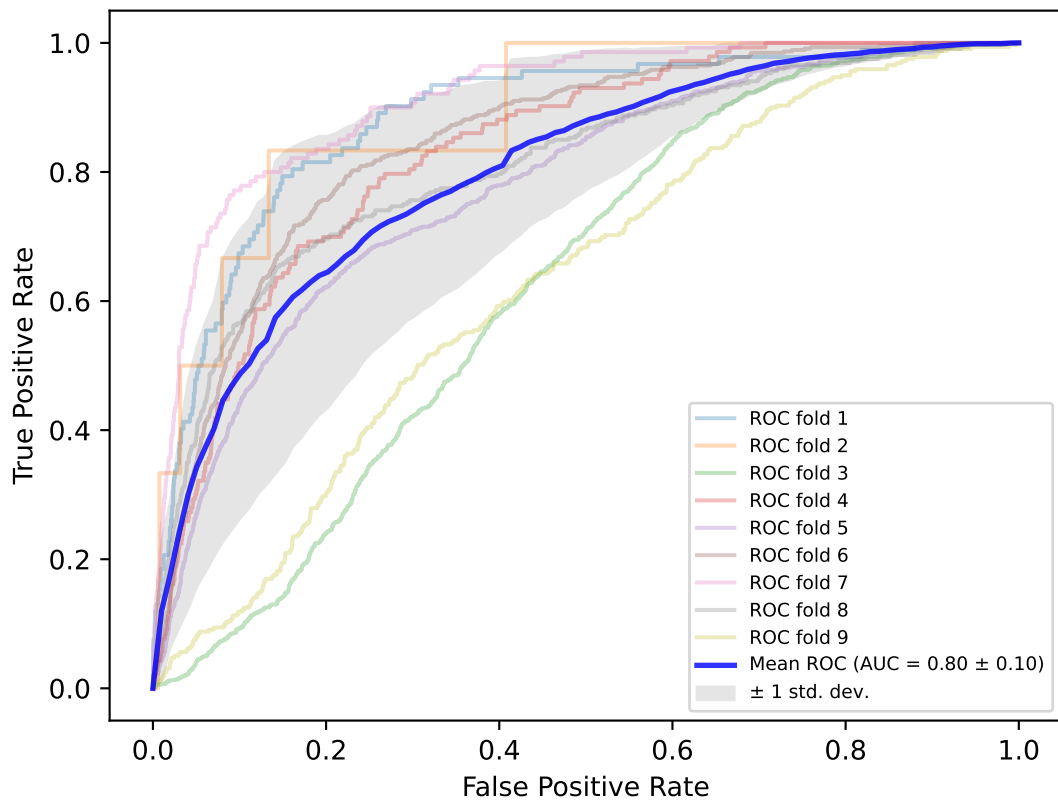

## Markov

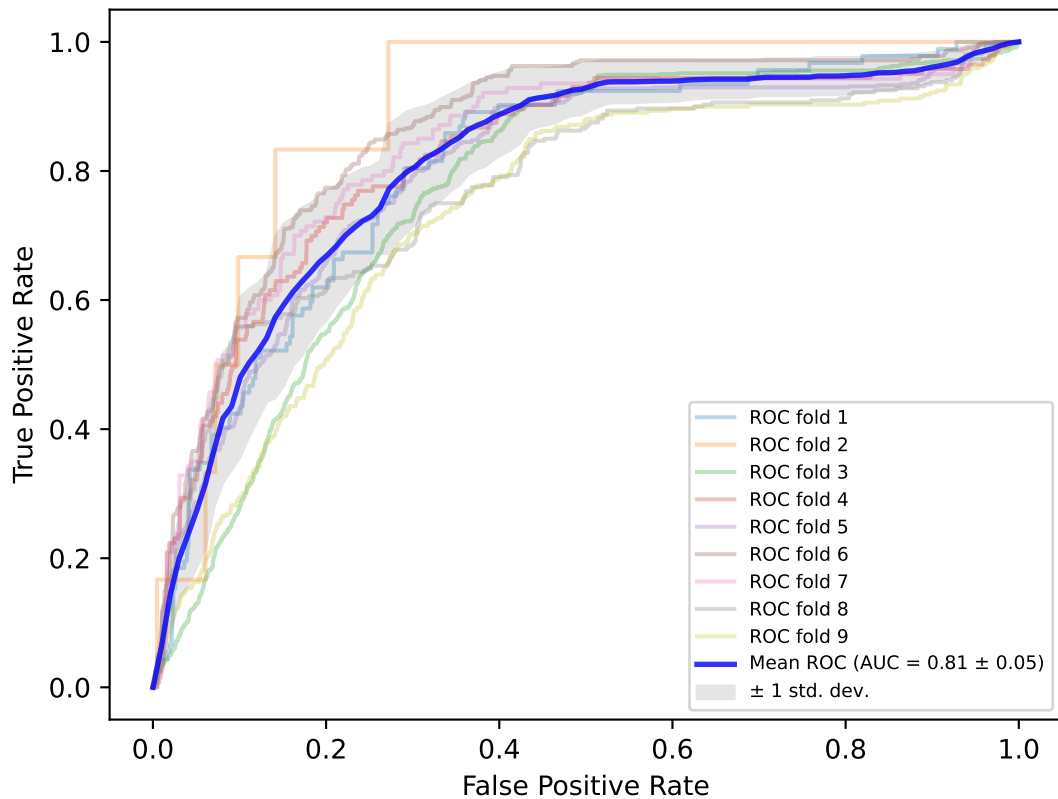

## PageRank

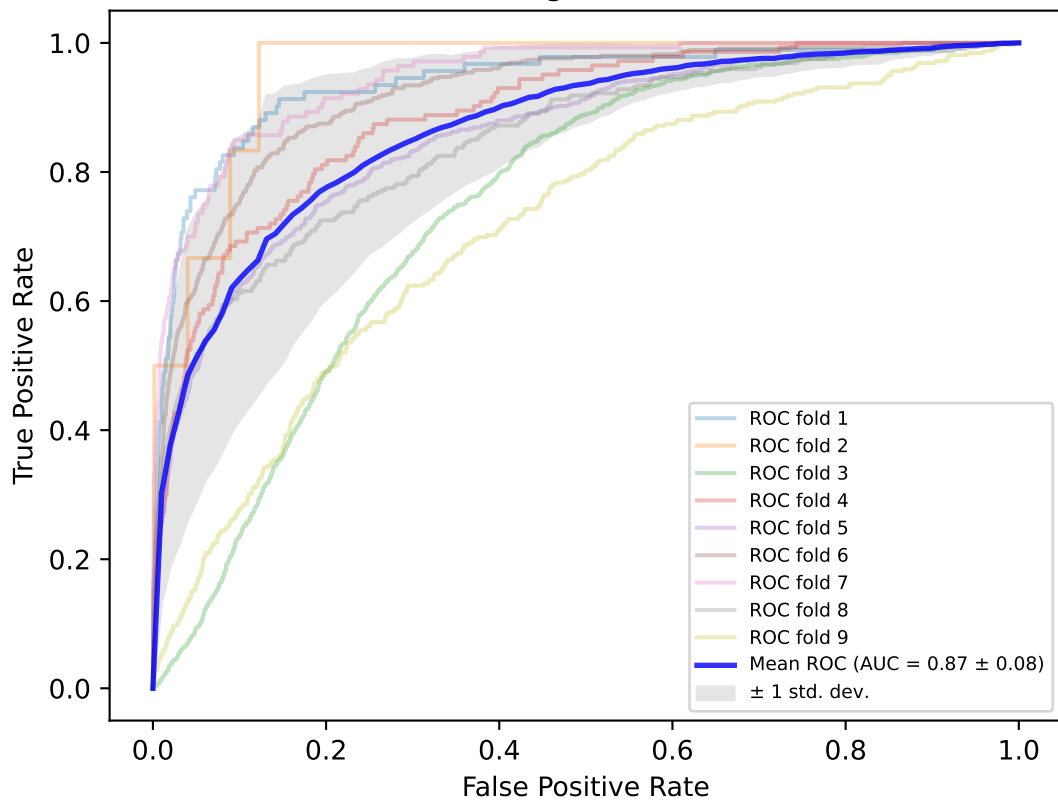

## BiPageRank

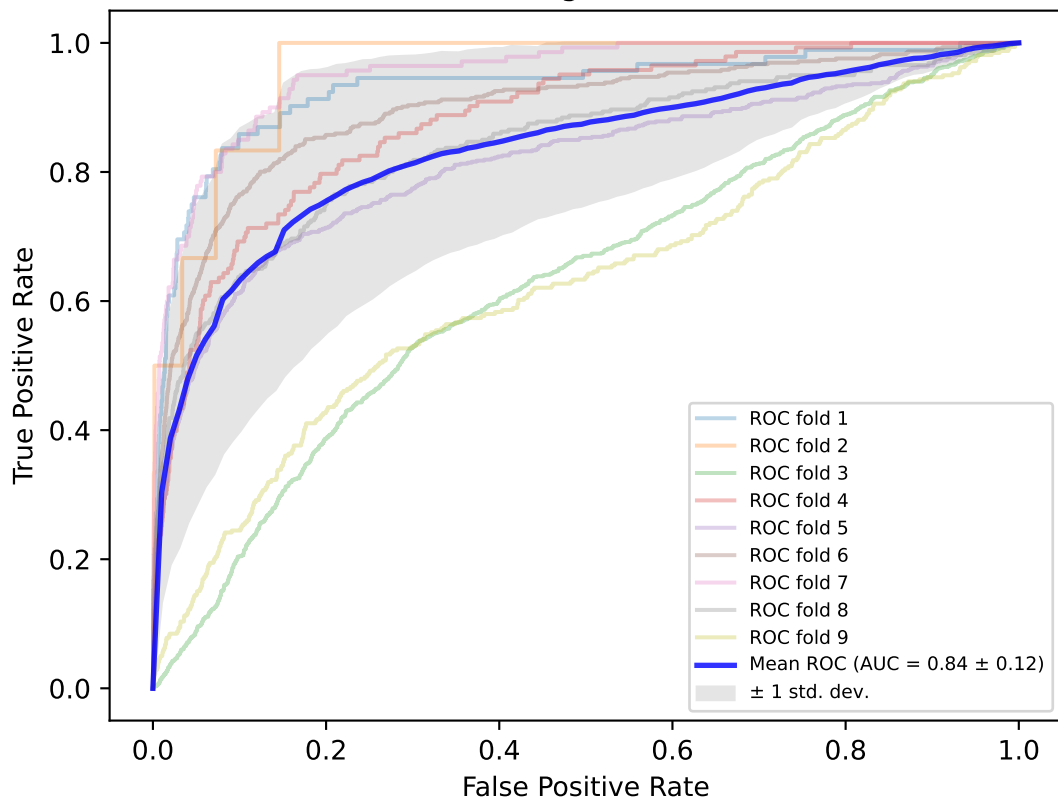

Supplement: Supplemental Information 3 [file peerj-cs-09-1686-s003.pdf]

## Classic

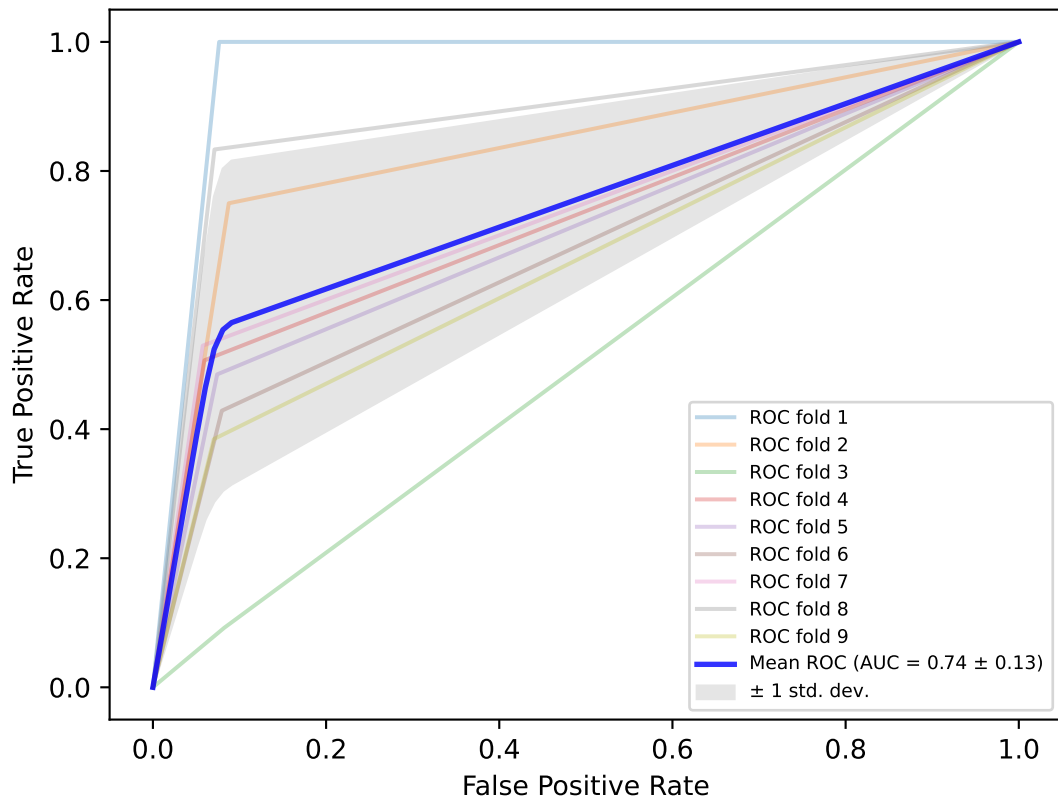

Count

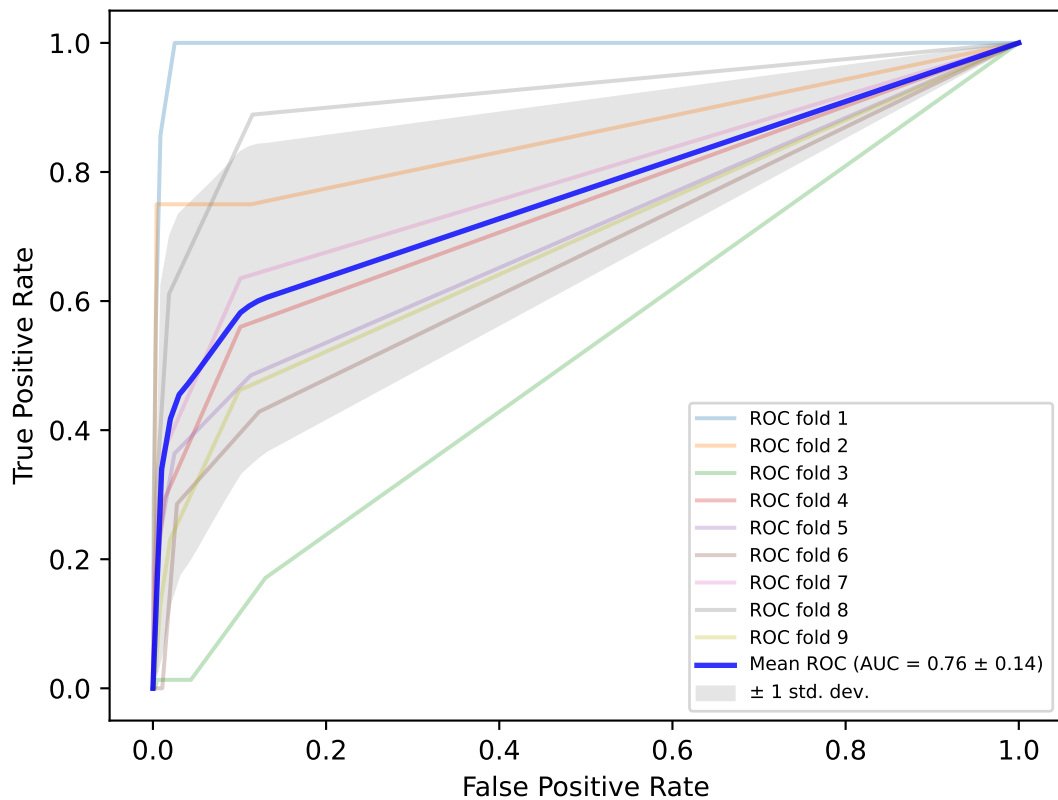

## Win-loss

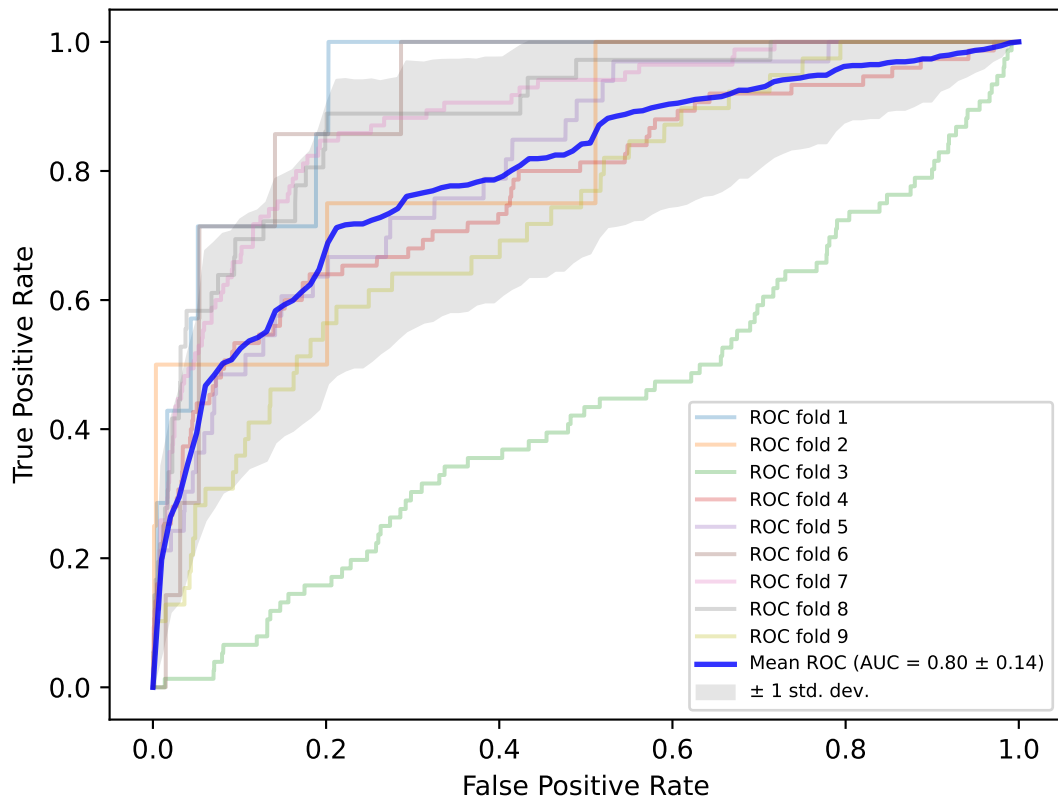

## Massey

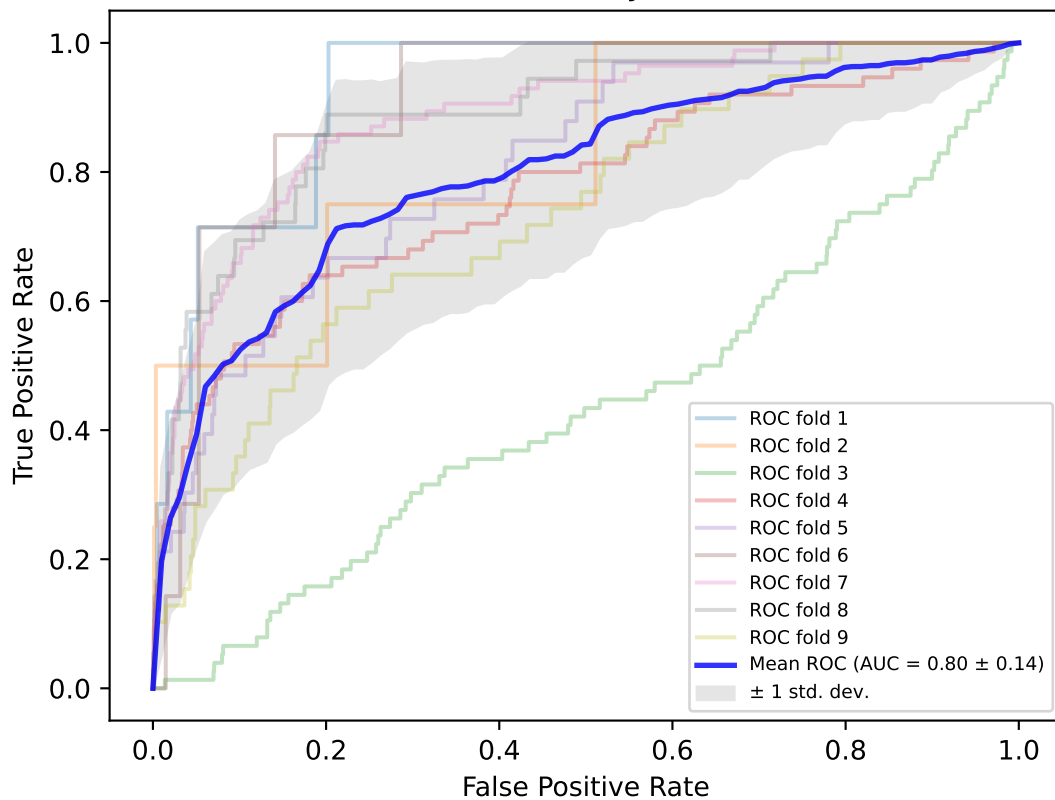

## Colley

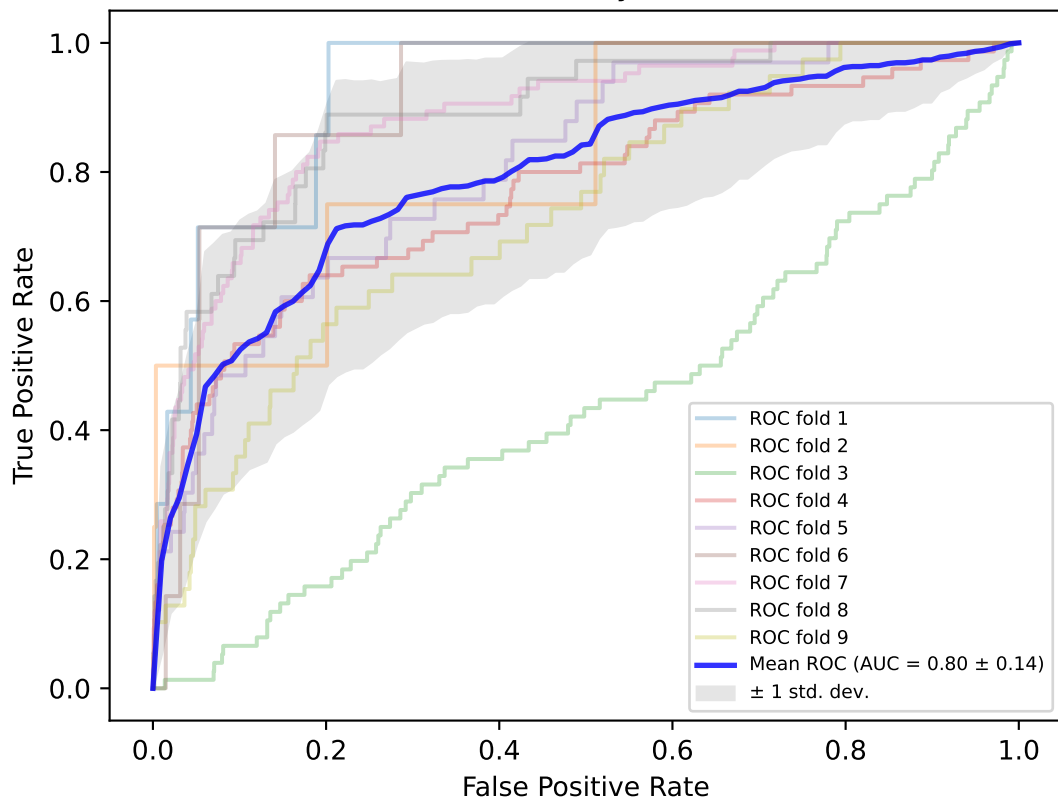

## Keener

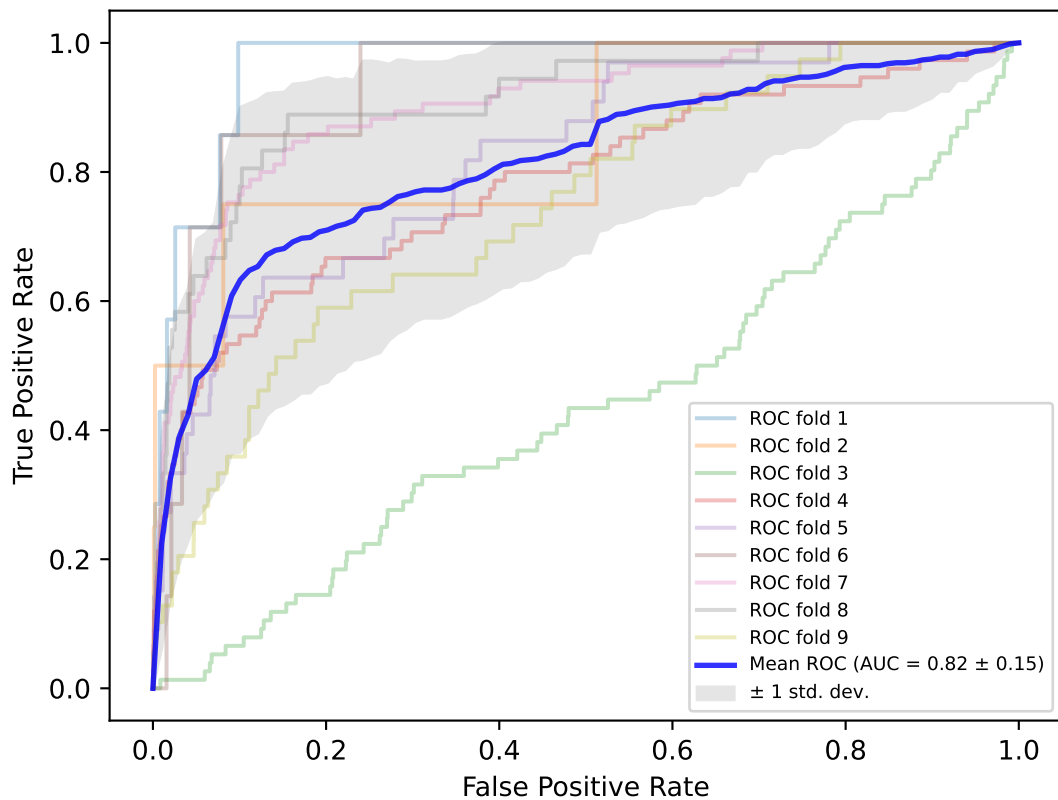

Elo

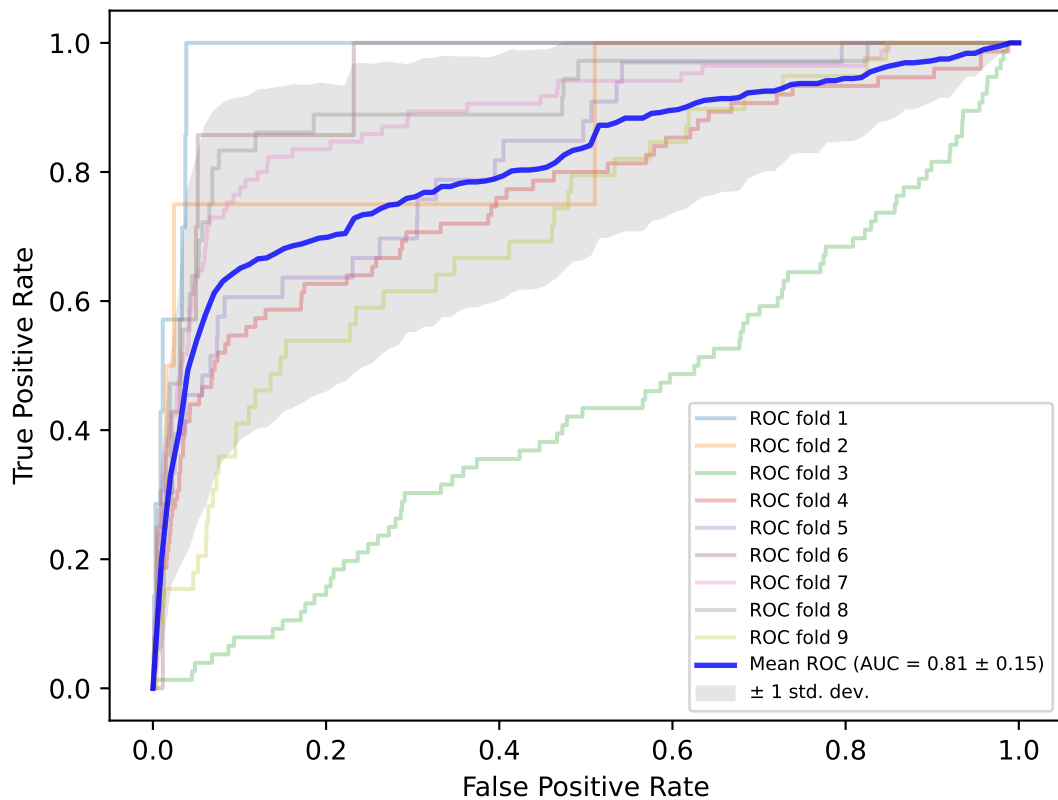

## Markov

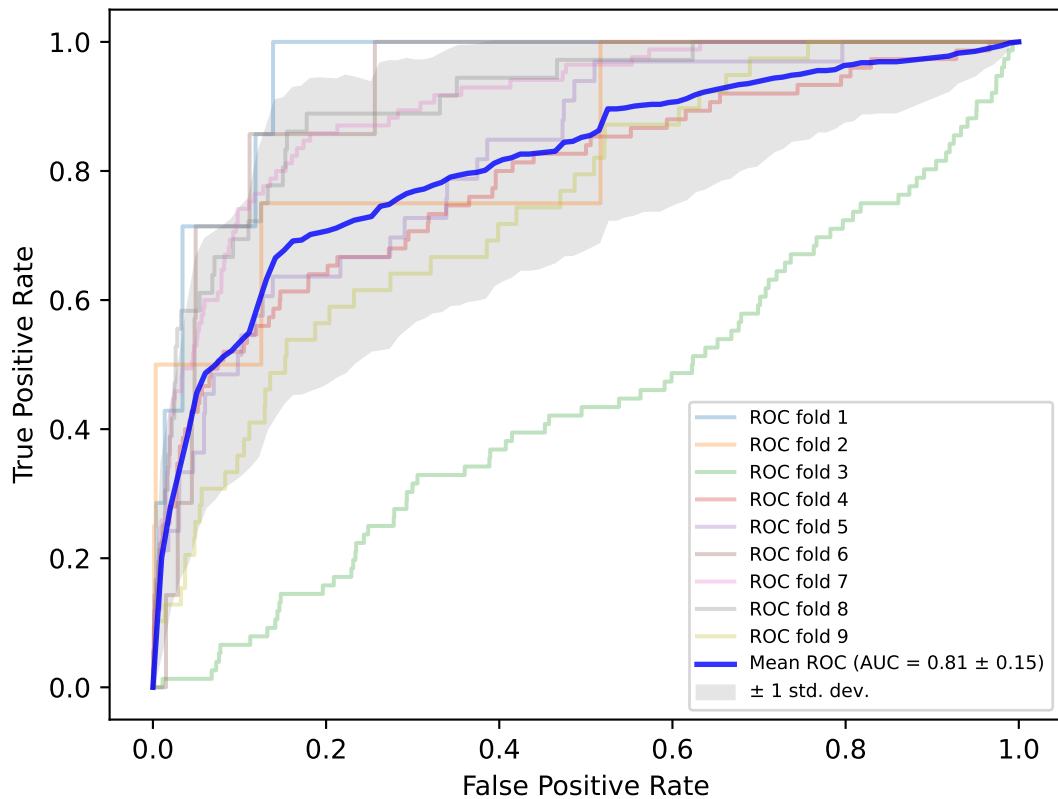

## PageRank

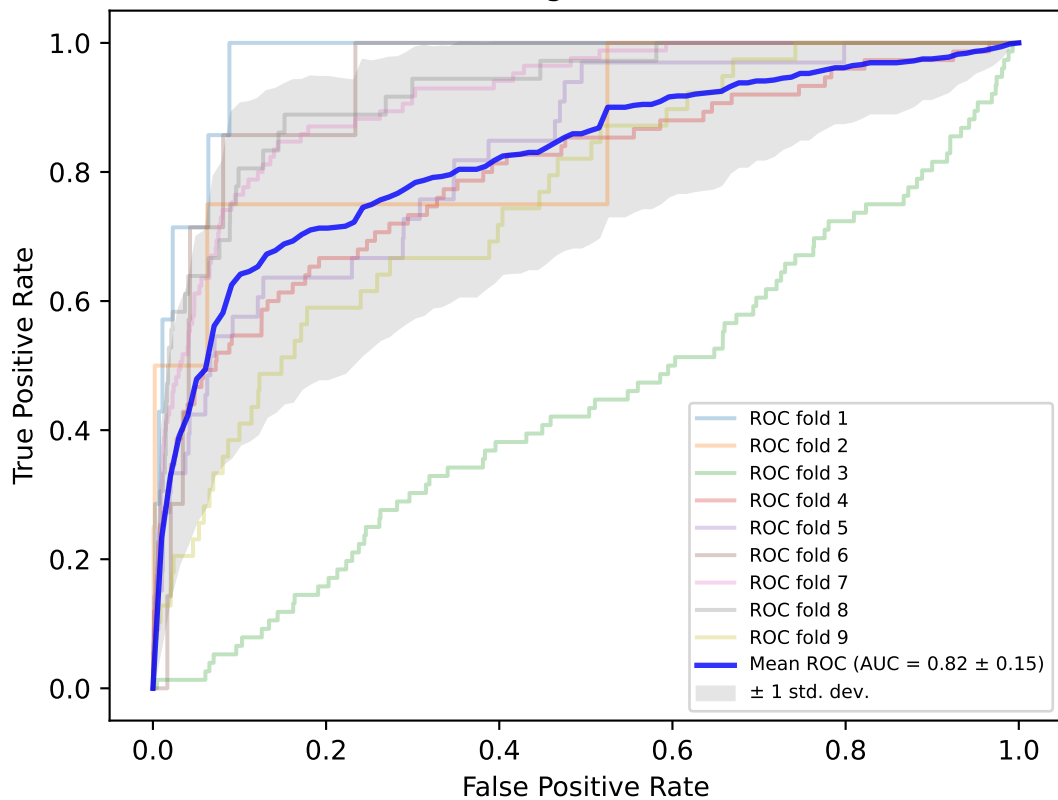

## BiPageRank

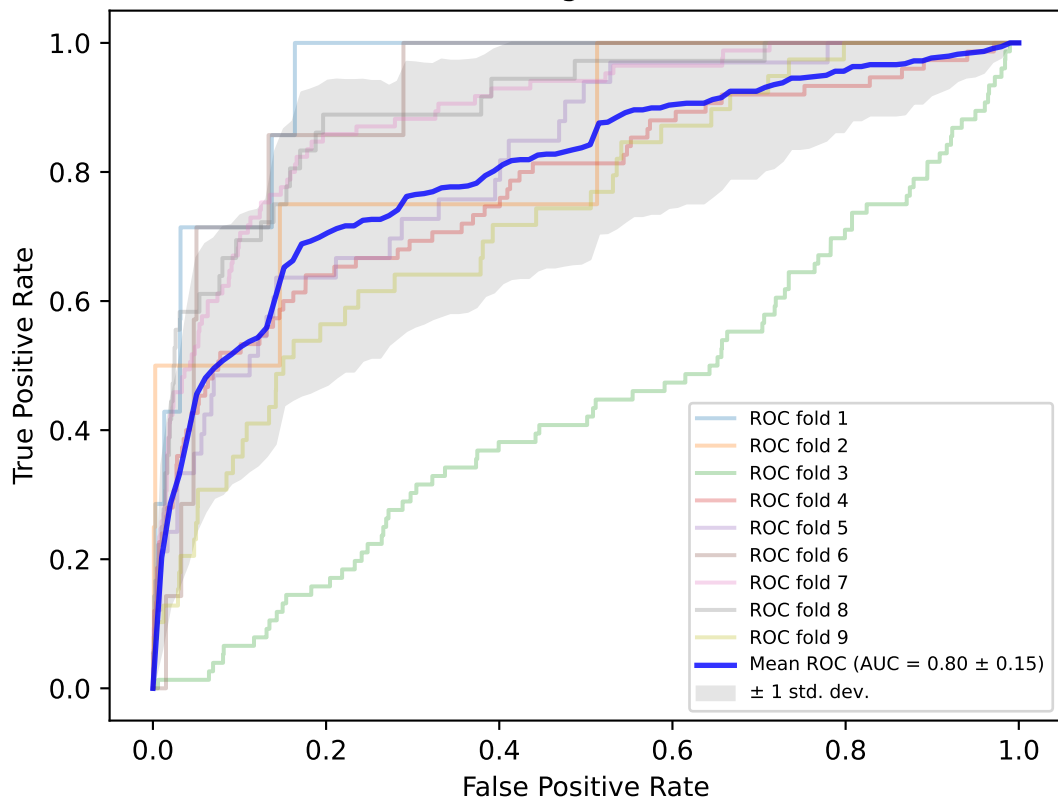

Supplement: Supplemental Information 4 [file peerj-cs-09-1686-s004.pdf]

## Classic

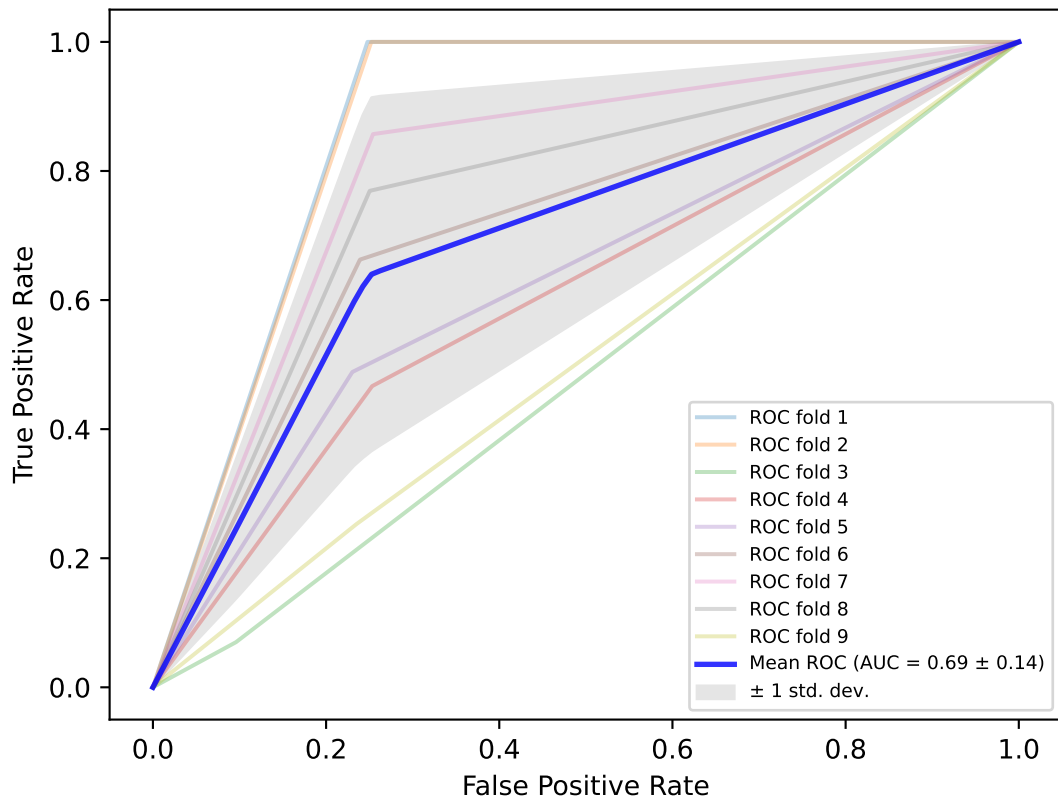

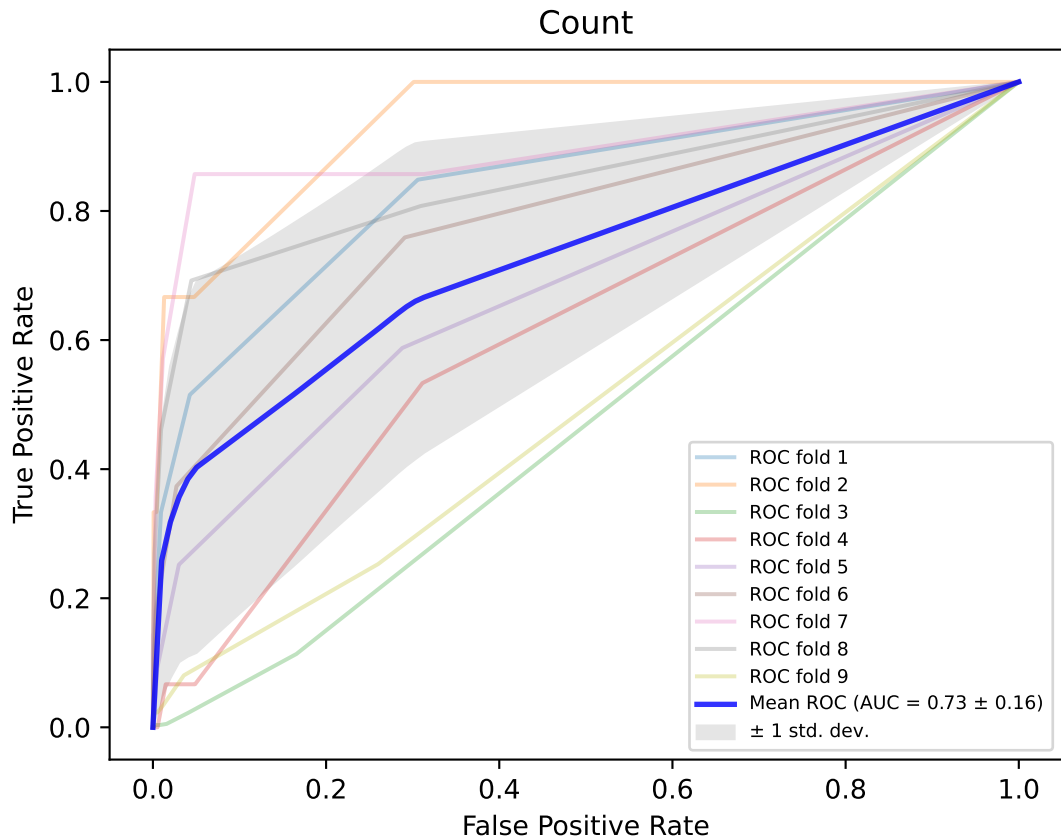

Win-loss

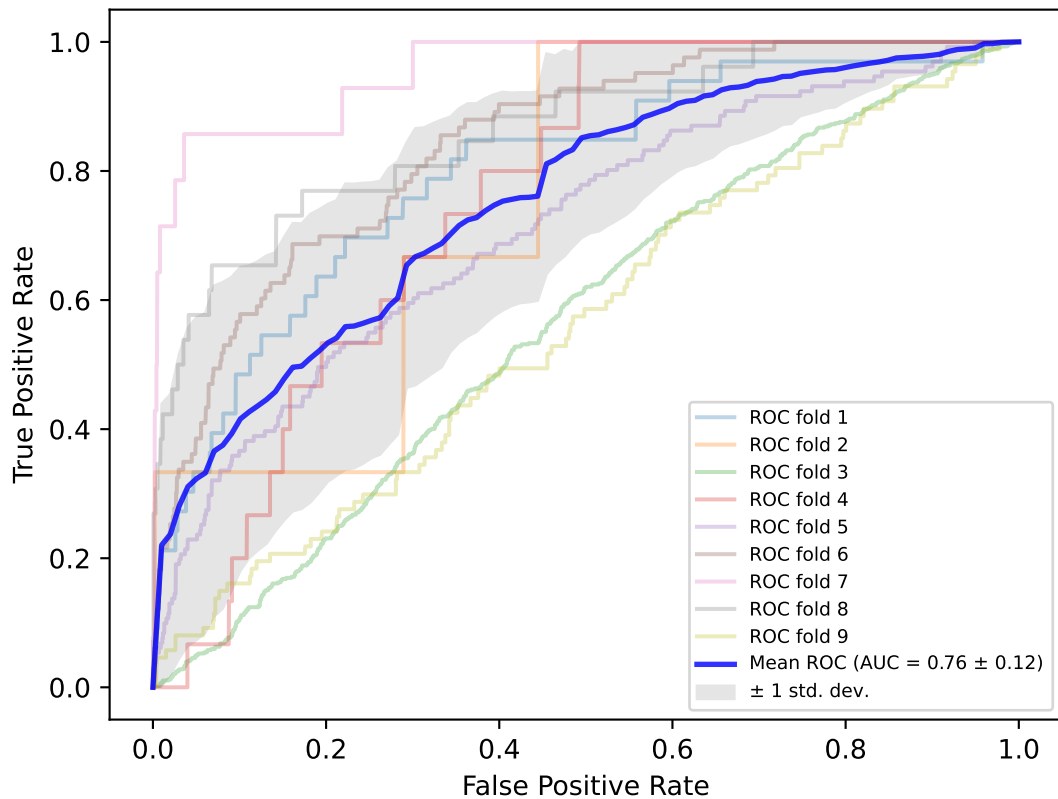

## Massey

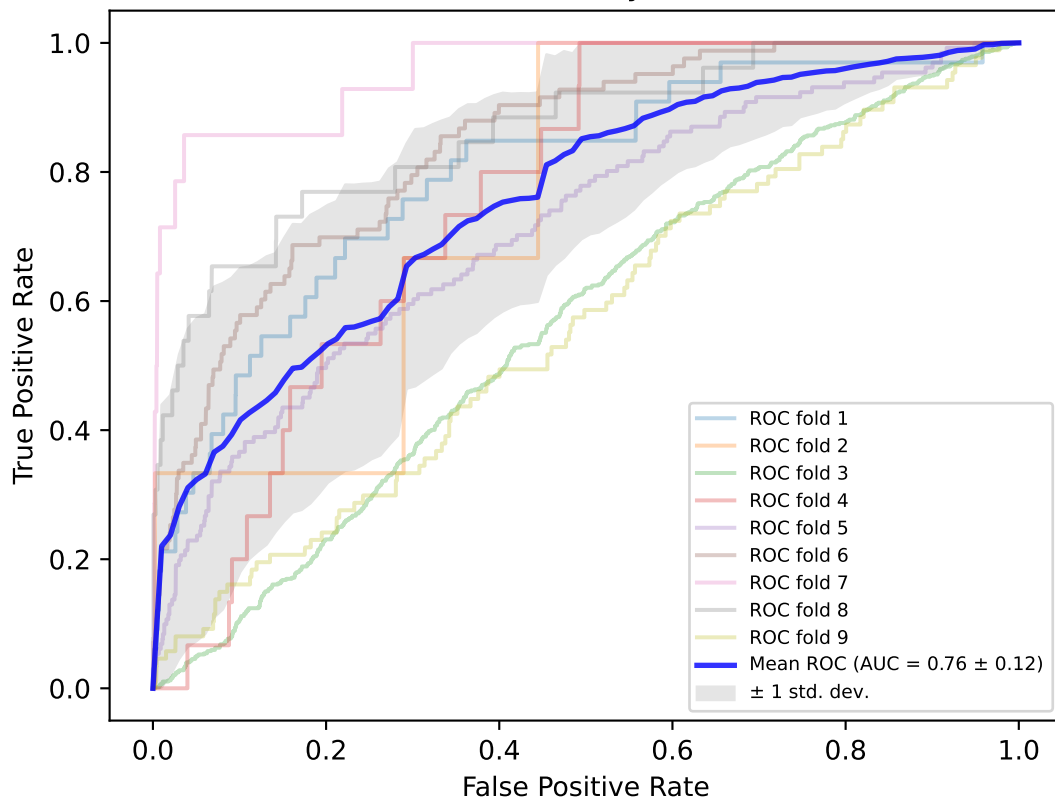

## Colley

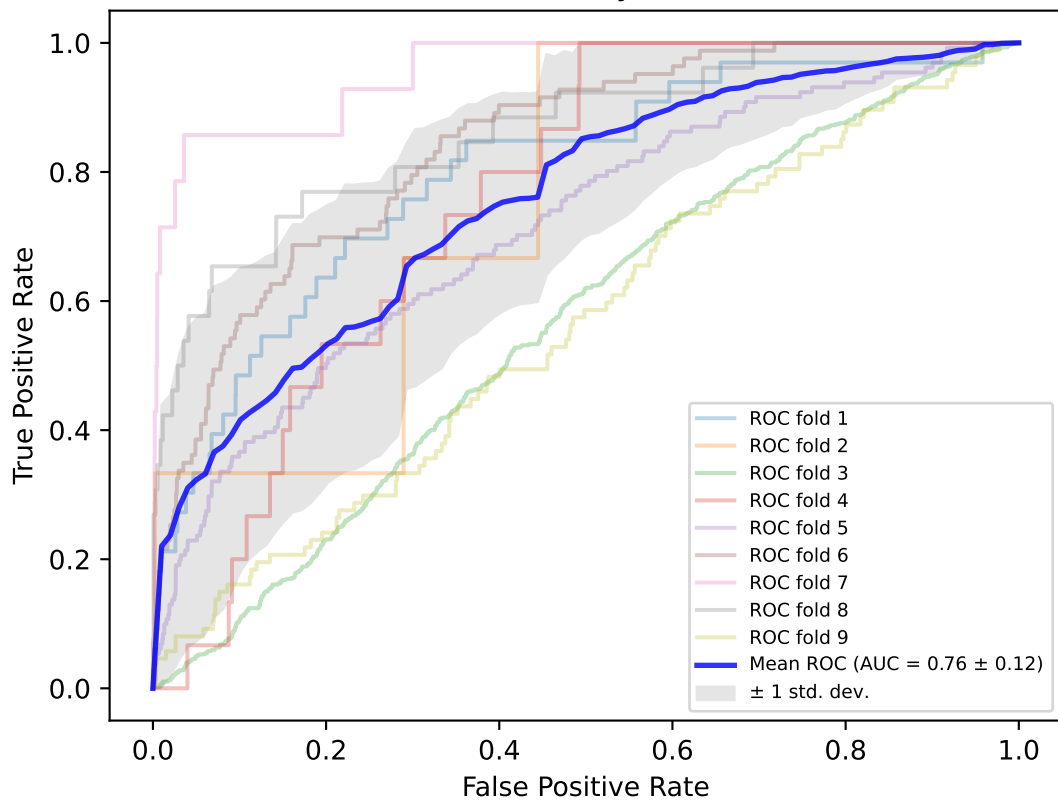

## Keener

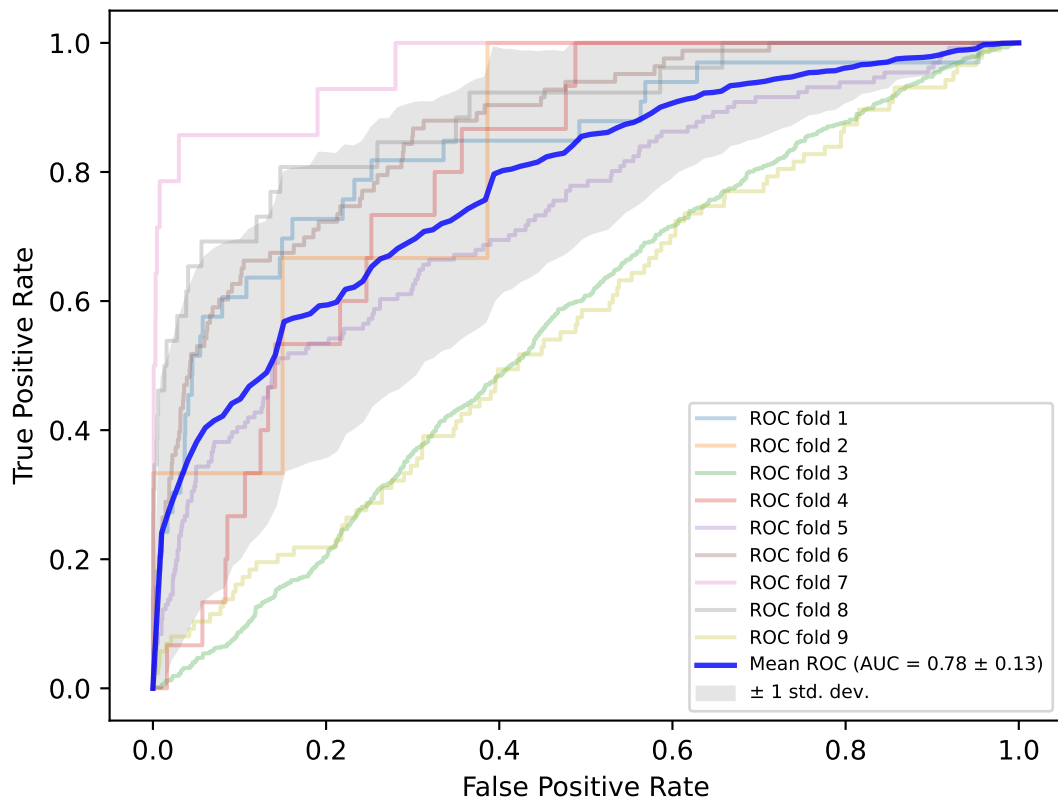

Elo

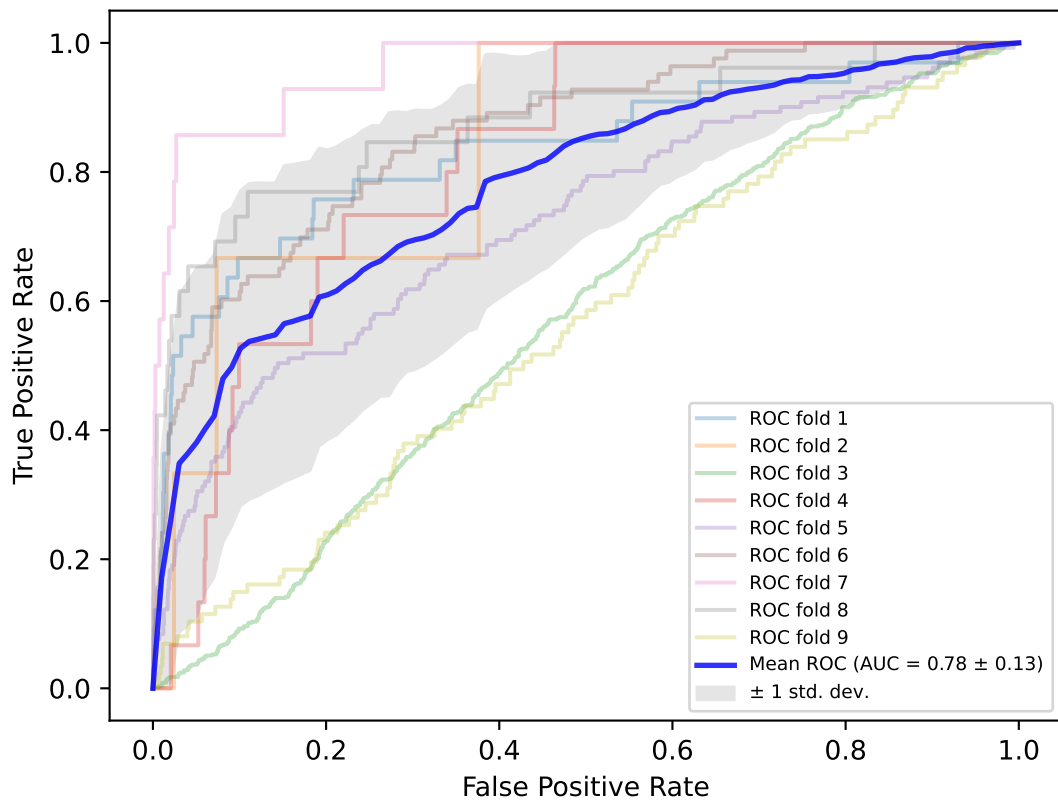

## Markov

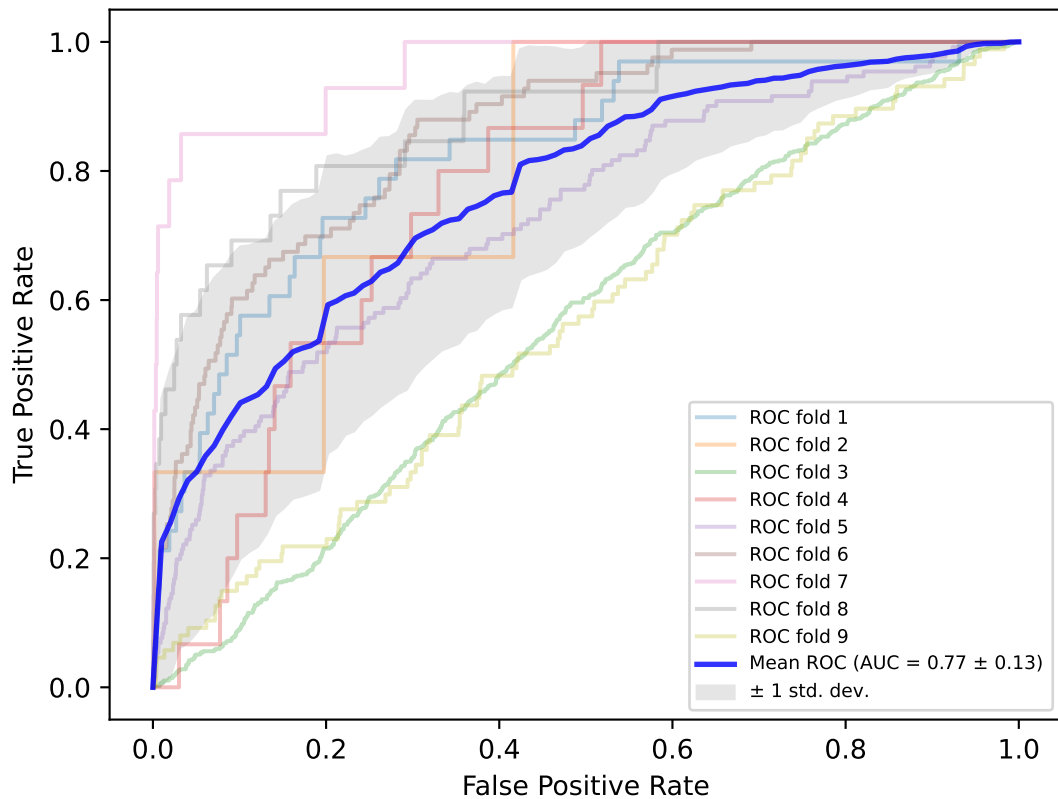

## PageRank

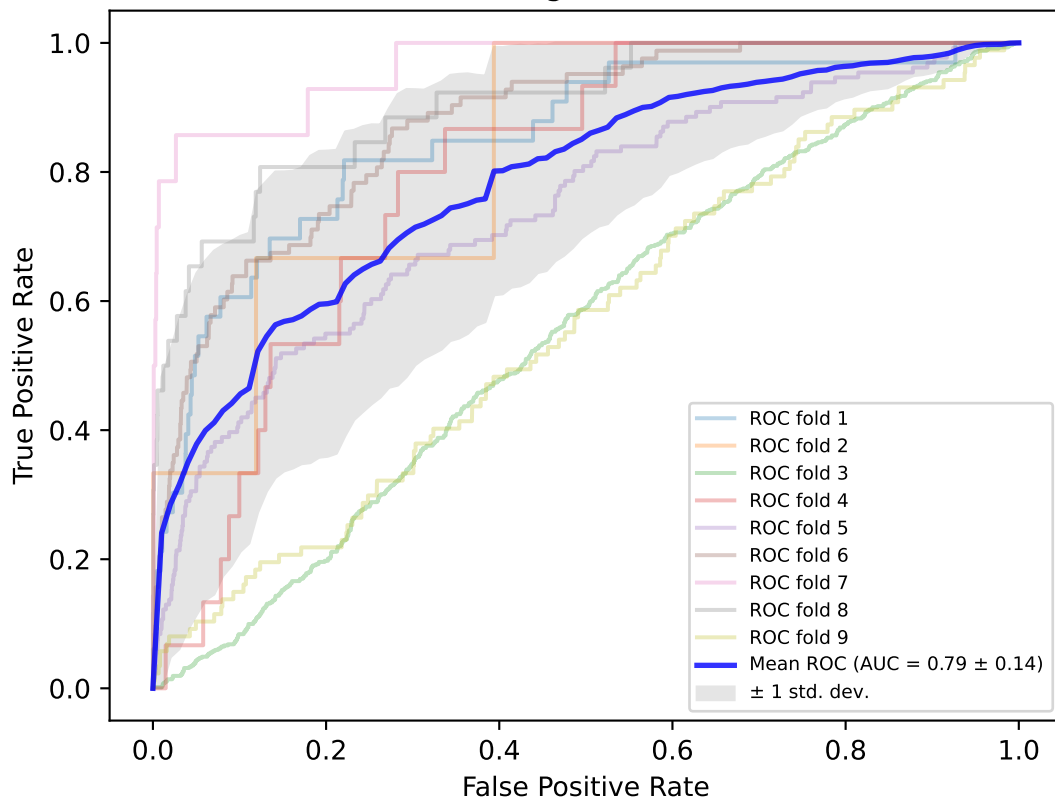

## BiPageRank

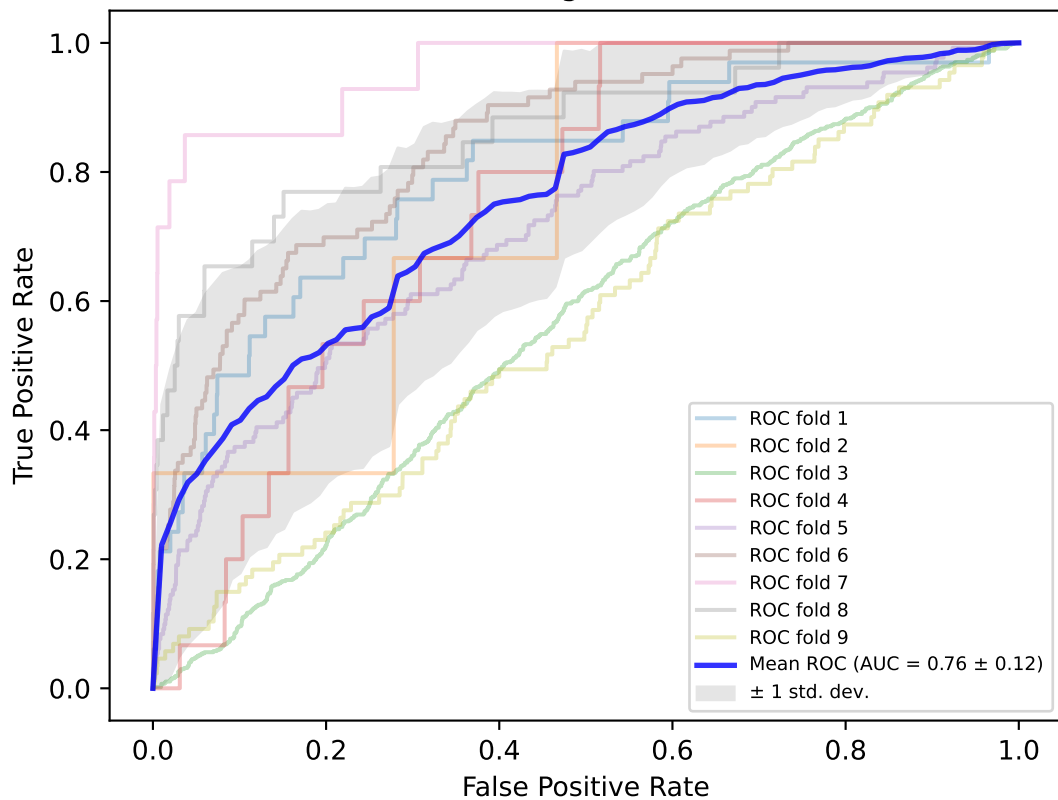

Supplement: Supplemental Information 5 [file peerj-cs-09-1686-s005.pdf]

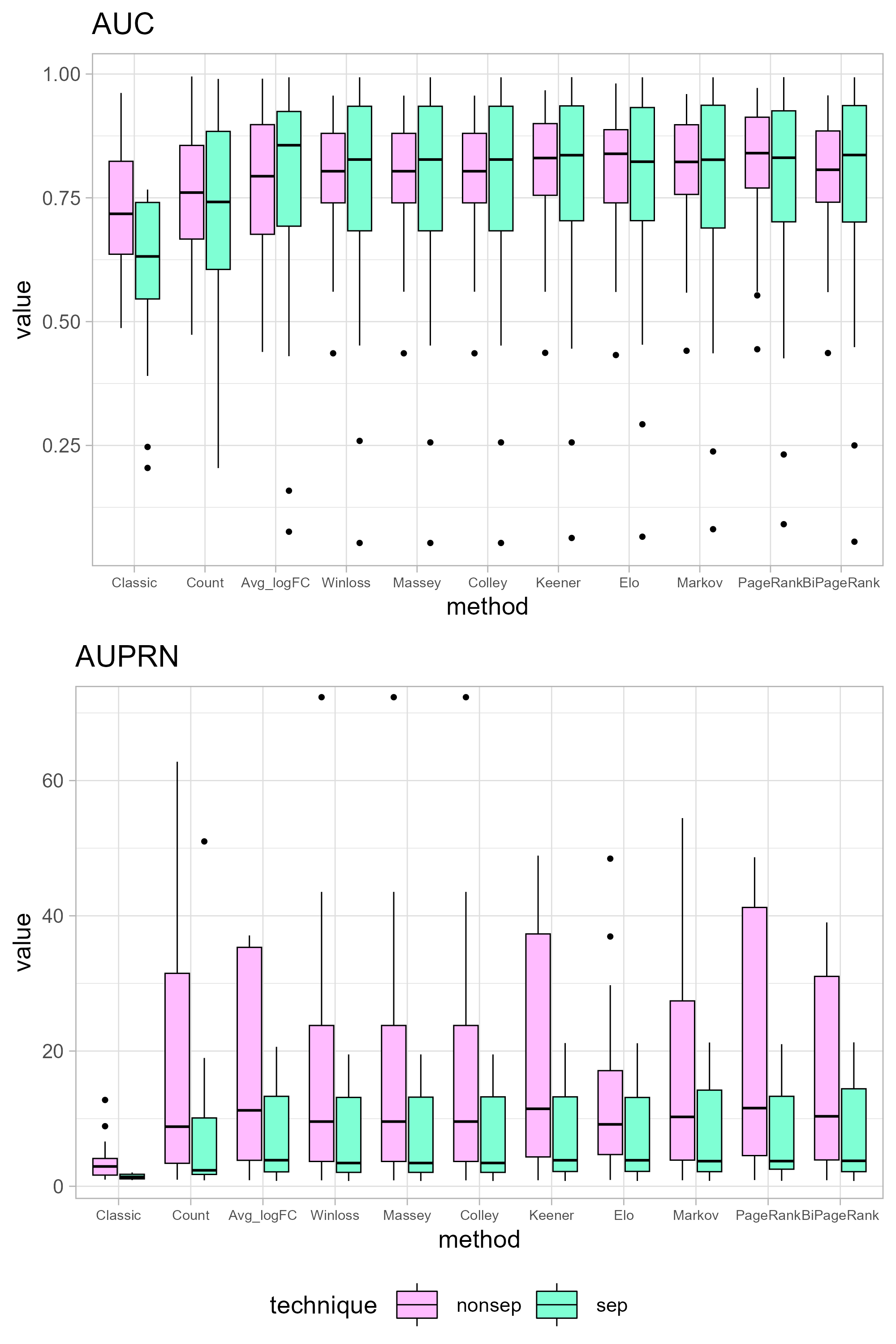

Supplement: Supplemental Information 6 [file peerj-cs-09-1686-s006.png]

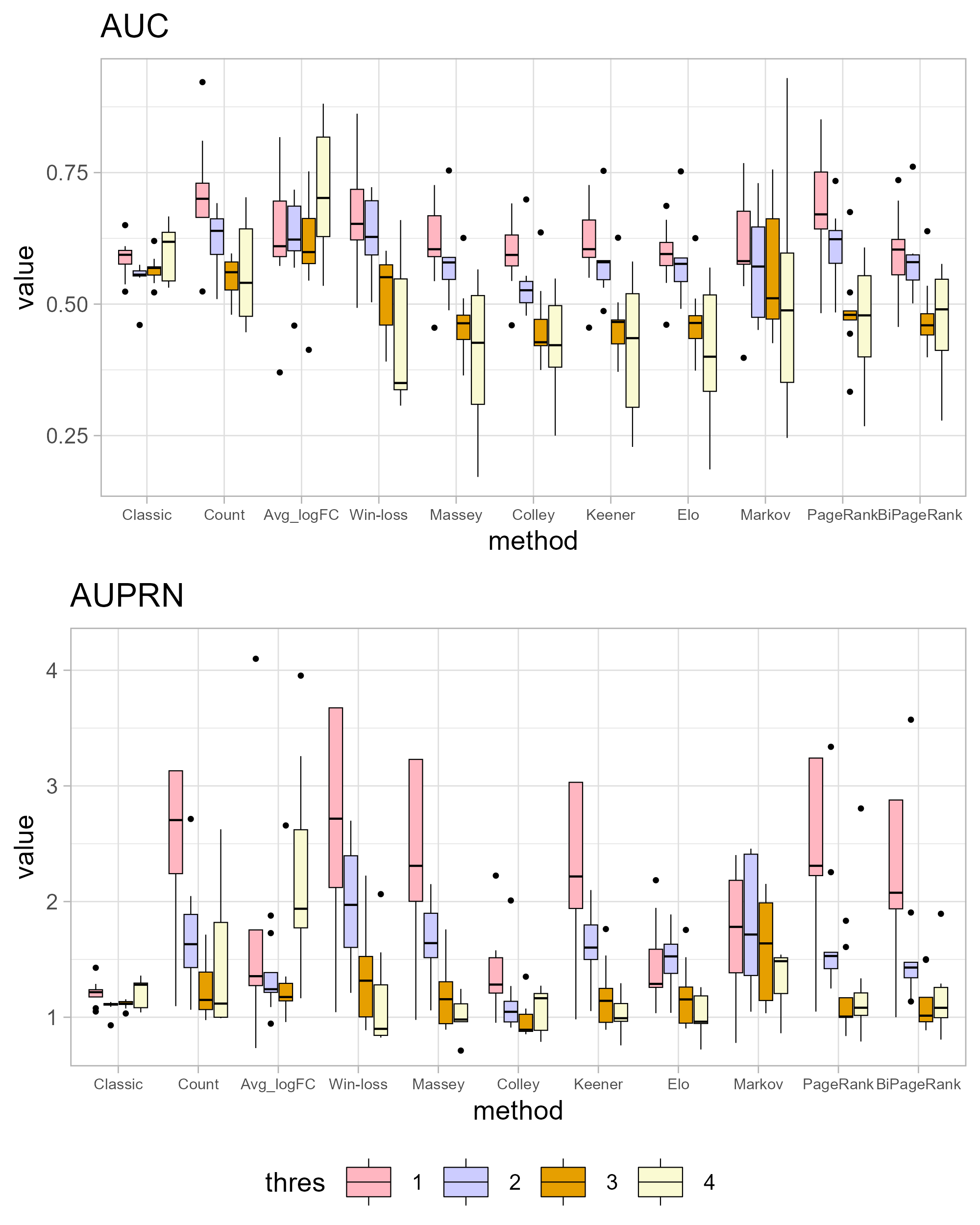

Supplement: Supplemental Information 7 [file peerj-cs-09-1686-s007.png]

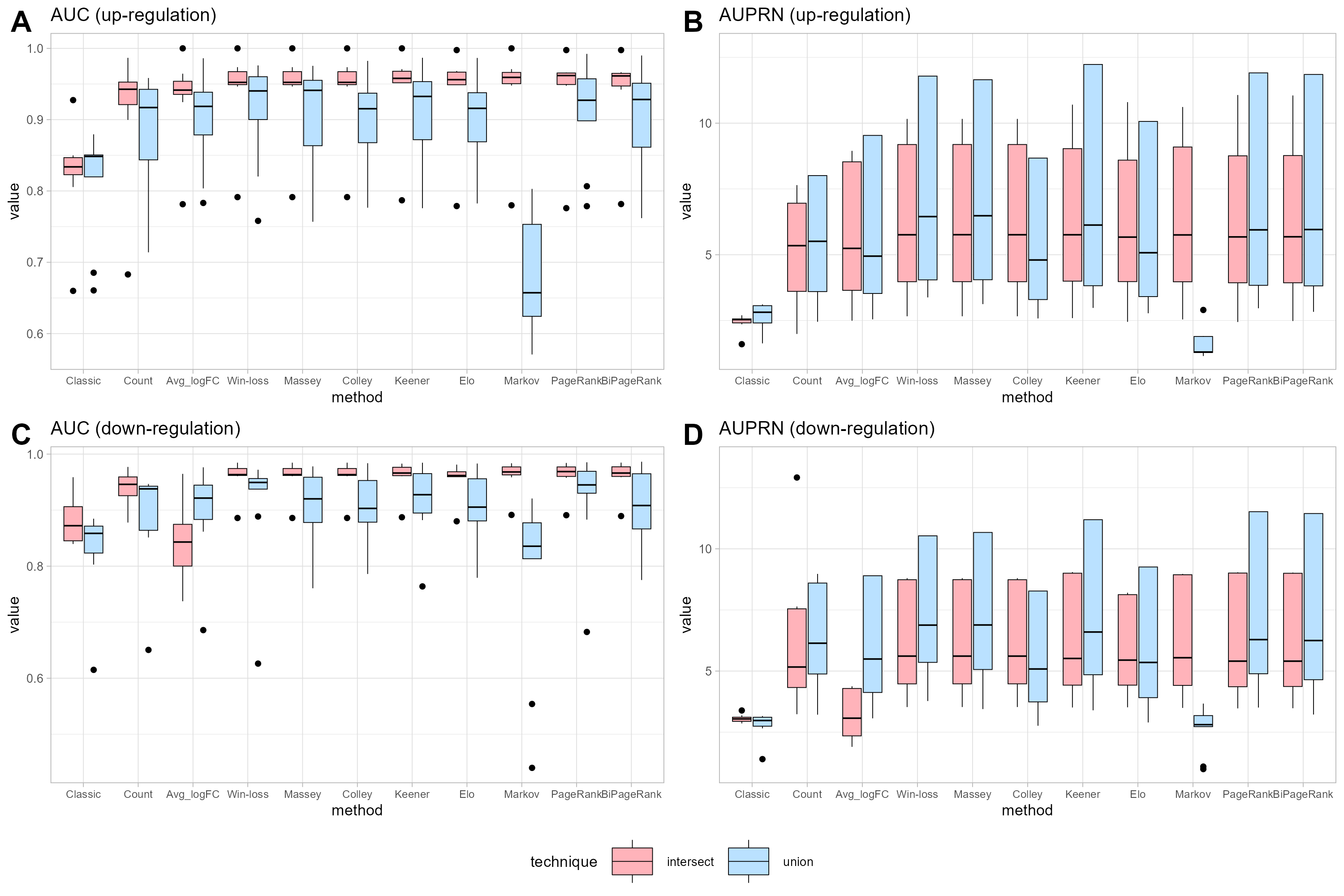

Supplement: Supplemental Information 8 [file peerj-cs-09-1686-s008.png]
