# Supplementary material for: GeneCompete: an integrative tool of a novel union algorithm with various ranking techniques for multiple gene expression data
Source: PeerJ Comput Sci. 2023 Nov 15;9:e1686. doi: 10.7717/peerj-cs.1686 (PMC10703088; doi:10.7717/peerj-cs.1686)
Supplement: Supplemental Information 9 [file peerj-cs-09-1686-s009.docx]

**Table S1** Dataset characteristics of HCM gene expression

| No. | GEO accession  no. | Platform | Technology | Case/control | Sample origins |
| --- | --- | --- | --- | --- | --- |
| 1 | GSE36961 | GPL15389 | Microarray | 106/39 | Rochester, USA |
| 2 | GSE32453 | GPL6104 | Microarray | 8/5 | Umeå, Sweden |
| 3 | GSE68316 | GPL20113 | Microarray | 7/5 | Chengdu, China |
| 4 | GSE1145 | GPL570 | Microarray | 5/11 | Cambridge, USA |
| 5 | GSE89714 | GPL11154 | RNA-Seq | 5/4 | Beijing, China |
| 6 | GSE130036 | GPL20795 | RNA-Seq | 28/9 | Beijing, China |
| 7 | GSE160997 | GPL11154 | RNA-Seq | 18/5 | Boston, USA |
| 8 | GSE180313 | GPL24676 | RNA-Seq | 13/7 | Stanford, USA |
| 9 | GSE141910 | GPL16791 | RNA-Seq | 28/166 | Philadelphia, USA |
