# Supplementary material for: GeneCompete: an integrative tool of a novel union algorithm with various ranking techniques for multiple gene expression data
Source: PeerJ Comput Sci. 2023 Nov 15;9:e1686. doi: 10.7717/peerj-cs.1686 (PMC10703088; doi:10.7717/peerj-cs.1686)
Supplement: Supplemental Information 10 [file peerj-cs-09-1686-s010.docx]

**Table S2** Dataset characteristics of MAQC and SEQC

| **No** | **GEO accession no.** | **Platform name** | **Platform** | **Technology** | **A/B** |
| --- | --- | --- | --- | --- | --- |
| 1 | GSE5350 | Agilent-012391 Whole Human Genome Oligo Microarray G4112A | GPL1708 | Microarray | 45/45 |
| 2 | GSE5350 | [HG-U133_Plus_2] Affymetrix Human Genome U133 Plus 2.0 Array | GPL570 | Microarray | 30/30 |
| 3 | GSE5350 | Sentrix Human-6 Expression BeadChip | GPL2507 | Microarray | 15/15 |
| 4 | GSE56457 | Illumina HumanHT-12 V4.0 expression beadchip | GPL10558 | Microarray | 6/6 |
| 5 | GSE56457 | [HuGene-2_0-st] Affymetrix Human Gene 2.0 ST Array | GPL17930 | Microarray | 4/4 |
| 6 | GSE56457 | GeneChip® PrimeView™ Human Gene Expression Array | GPL16043 | Microarray | 4/4 |
| 7 | GSE47774 | AB 5500 Genetic Analyzer (Homo sapiens) | GPL16558 | RNA-Seq | 190/190 |
| 8 | GSE47774 | Illumina HiSeq 2000 (Homo sapiens) | GPL11154 | RNA-Seq | 423/423 |
| 9 | GSE48016 | Agilent-039825 SEQCboku1Mv1 | GPL17298 | Microarray | 4/4 |
