# Supplementary material for: GeneCompete: an integrative tool of a novel union algorithm with various ranking techniques for multiple gene expression data
Source: PeerJ Comput Sci. 2023 Nov 15;9:e1686. doi: 10.7717/peerj-cs.1686 (PMC10703088; doi:10.7717/peerj-cs.1686)
Supplement: Supplemental Information 11 [file peerj-cs-09-1686-s011.docx]

**Table S3** The *logFC* of top 10 genes identified by using PageRank method with intersection strategy in up-regulated case

|  | **Genes** | **GSE**  **36961** | **GSE**  **32453** | **GSE**  **68316** | **GSE**  **1145** | **GSE**  **89714** | **GSE**  **130036** | **GSE**  **160997** | **GSE**  **180313** | **GSE**  **141910** |
| --- | --- | --- | --- | --- | --- | --- | --- | --- | --- | --- |
| 1 | COL16A1 | 0.8355 | 0.9290 | 0.8577 | 0.5653 | 1.5240 | 1.2193 | 0.7012 | 1.3486 | 1.5328 |
| 2 | NTNG2 | 0.1456 | 3.0588 | 0.4744 | 1.0089 | 1.3253 | 0.8118 | 0.8175 | 2.2584 | 1.4057 |
| 3 | MBP | 0.9629 | 0.6855 | 0.1439 | 1.1150 | 1.0049 | 0.9150 | 0.4059 | 1.3553 | 0.8818 |
| 4 | PROS1 | 1.4721 | 2.0332 | -0.4914 | 1.0006 | 1.5247 | 1.2672 | 0.2401 | 1.8796 | 0.5798 |
| 5 | KLHL34 | 0.8617 | 1.6786 | 0.2277 | 1.3441 | 0.7384 | 0.5671 | 0.5955 | 0.7445 | 0.5064 |
| 6 | EDNRA | 0.9115 | 0.3113 | 0.5003 | 0.9205 | 1.3701 | 1.0382 | 0.3609 | 1.2652 | 0.4651 |
| 7 | FNDC1 | 0.7572 | 0.0522 | 1.0597 | 2.2267 | 1.7622 | -0.2189 | 1.3893 | 3.1515 | 2.7176 |
| 8 | PIK3IP1 | 1.3552 | 0.8368 | -0.5365 | 0.6127 | 1.6874 | 0.6016 | 0.5073 | 1.5099 | 0.9249 |
| 9 | MAP4 | 0.6822 | 0.9527 | 0.6598 | 0.1606 | 1.1685 | 0.9606 | 0.2231 | 0.8098 | 0.4787 |
| 10 | ISLR | 0.3315 | 0.2422 | 0.3243 | 1.5253 | 2.1753 | 0.1402 | 0.2035 | 1.6016 | 1.881 |
