# Supplementary material for: GeneCompete: an integrative tool of a novel union algorithm with various ranking techniques for multiple gene expression data
Source: PeerJ Comput Sci. 2023 Nov 15;9:e1686. doi: 10.7717/peerj-cs.1686 (PMC10703088; doi:10.7717/peerj-cs.1686)
Supplement: Supplemental Information 12 [file peerj-cs-09-1686-s012.docx]

**Table S4** The *logFC* of top 10 genes identified by using PageRank method with intersection strategy in down-regulated case

|  | **Genes** | **GSE**  **36961** | **GSE**  **32453** | **GSE**  **68316** | **GSE**  **1145** | **GSE**  **89714** | **GSE**  **130036** | **GSE**  **160997** | **GSE**  **180313** | **GSE**  **141910** |
| --- | --- | --- | --- | --- | --- | --- | --- | --- | --- | --- |
| 1 | HOPX | -1.5082 | -1.3416 | -2.1047 | -1.6776 | -1.4506 | -1.2131 | -1.7880 | -2.8333 | -2.9493 |
| 2 | MYH6 | -1.9145 | -1.7706 | -0.0860 | -1.8508 | -0.3802 | -1.1571 | -2.6361 | -3.5735 | -2.7432 |
| 3 | NAMPT | -0.8779 | -0.9496 | -0.6204 | -1.7178 | -0.2884 | -0.7655 | -1.4016 | -0.5644 | -1.0953 |
| 4 | SLC2A1 | -0.7697 | -0.9005 | -0.4203 | -0.6840 | -0.4766 | -1.0718 | -1.1476 | -0.9431 | -1.0573 |
| 5 | C3 | -0.4181 | -1.6411 | 0.3463 | -0.7941 | -1.5797 | -0.8621 | -2.0651 | -1.5695 | -1.1150 |
| 6 | LDHA | -0.9360 | -0.3878 | -0.5793 | -0.4904 | -0.8888 | -1.3913 | -1.1865 | -1.0894 | -0.7428 |
| 7 | LRRN3 | -0.3612 | -0.5880 | -1.1393 | -0.2251 | -1.7265 | -0.6578 | -1.9850 | -1.8783 | -1.0083 |
| 8 | IL18R1 | -0.7906 | -1.3631 | -0.7033 | -0.5974 | -0.3055 | -0.6283 | -1.7606 | -1.5127 | -1.7222 |
| 9 | EIF4EBP1 | -0.1522 | -0.8284 | -0.8651 | -0.8734 | -0.1906 | -0.9766 | -1.7057 | -1.2022 | -1.1464 |
| 10 | S1PR3 | -1.0088 | -0.5460 | 0.3874 | -0.5198 | -0.6227 | -1.2972 | -1.6569 | -1.1407 | -1.4397 |
