# Supplementary material for: GeneCompete: an integrative tool of a novel union algorithm with various ranking techniques for multiple gene expression data
Source: PeerJ Comput Sci. 2023 Nov 15;9:e1686. doi: 10.7717/peerj-cs.1686 (PMC10703088; doi:10.7717/peerj-cs.1686)
Supplement: Supplemental Information 13 [file peerj-cs-09-1686-s013.docx]

Table S5 Top-ranking genes identifies by different methods (up-regulation)

|  | **Win-loss** | **Massey** | **Colley** | **Keener** | **Elo** | **Markov** | **PageRank** | **BiPageRank** |
| --- | --- | --- | --- | --- | --- | --- | --- | --- |
| 1 | SFRP4 | SFRP4 | BMPR1AP1 | SFRP4 | CA3 | KLHL34 | SLITRK4 | SLITRK4 |
| 2 | SLITRK4 | SLITRK4 | SORBS2-AS1 | SLITRK4 | CENPA | XAF1 | SFRP4 | SFRP4 |
| 3 | CA3 | DIO2 | MKRN5P | DIO2 | PENK | COPS7B | CA3 | CA3 |
| 4 | DIO2 | THBS4 | SLC2A3P2 | CA3 | LEPREL1 | TMCO3 | FRZB | FRZB |
| 5 | THBS4 | MXRA5 | MYOM3-AS1 | THBS4 | LOC100509457 | ARVCF | MXRA5 | MXRA5 |
| 6 | MXRA5 | ST8SIA2 | NSA2P5 | MXRA5 | IGLC1 | MDM4 | SMOC2 | SMOC2 |
| 7 | FMOD | AEBP1 | GUSBP9 | FMOD | SFRP4 | CTNS | THBS4 | THBS4 |
| 8 | AEBP1 | FMOD | NUTM2G | SMOC2 | LOC102723648 | MAP4 | FNDC1 | PENK |
| 9 | FAP | SMOC2 | LARGE-AS1 | AEBP1 | SLITRK4 | BCAM | FMOD | FMOD |
| 10 | SMOC2 | CA3 | H1-9P | FAP | CAPN6 | EPS8L1 | DIO2 | DIO2 |
