# Supplementary material for: GeneCompete: an integrative tool of a novel union algorithm with various ranking techniques for multiple gene expression data
Source: PeerJ Comput Sci. 2023 Nov 15;9:e1686. doi: 10.7717/peerj-cs.1686 (PMC10703088; doi:10.7717/peerj-cs.1686)
Supplement: Supplemental Information 14 [file peerj-cs-09-1686-s014.docx]

Table S6 Top-ranking genes identifies by different methods (down-regulation)

|  | **Win-loss** | **Massey** | **Colley** | **Keener** | **Elo** | **Markov** | **PageRank** | **BiPageRank** |
| --- | --- | --- | --- | --- | --- | --- | --- | --- |
| 1 | HOPX | HOPX | RP11-481J2.2 | HOPX | SERPINA3 | COPS6 | FCN3 | FCN3 |
| 2 | FCN3 | FCN3 | RP11-766F14.2 | FCN3 | LOC285556 | TMEM203 | CORIN | CORIN |
| 3 | SMTNL2 | SMTNL2 | TRAC | SMTNL2 | FCN3 | SLC25A42 | HOPX | HOPX |
| 4 | CORIN | CORIN | RP1-85F18.5 | CORIN | HOPX | FLOT2 | MYH6 | SERPINA3 |
| 5 | LSAMP | LSAMP | RP11-109L13.1 | LSAMP | TUBA3E | MTFMT | SERPINA3 | MYH6 |
| 6 | FKBP5 | MYH6 | RP11-6F2.6 | FKBP5 | AXUD1 | SMYD3 | TUBA3E | TUBA3E |
| 7 | CD163 | C3 | AQP7P4 | CD163 | CD209 | PDHA1 | CD163 | SMTNL2 |
| 8 | AZGP1 | CCL2 | AC007126.1 | METTL7B | METTL7B | DGAT1 | SMTNL2 | CD163 |
| 9 | RARRES1 | CD163 | CTD-2281M20.1 | MYH6 | LINC02809 | ACSS2 | CCL2 | CCL2 |
| 10 | CCL2 | AZGP1 | RP11-256I23.3 | RARRES1 | POLG-DT | EVI5L | RARRES1 | RARRES1 |
